# Replicative and stress-induced premature senescence distinctively affect the endothelial anticoagulation capacity

**DOI:** 10.1371/journal.pone.0351140

**Published:** 2026-06-09

**Authors:** Akiko Katayama, Koji Ikeda, Tomoya Kitani, Ekura Yamazaki, Daisuke Ueno, Fumiaki Ito, Satoaki Matoba

**Affiliations:** 1 Department of Cardiology, Kyoto Prefectural University of Medicine, 465 Kajii, Kawaramachi-Hirokoji, ‌‌Kamigyo, Kyoto, Japan; 2 Department of Internal Medicine, Ikeda Hospital, 1-9-28 Hoji, Higashi-osaka, ‌‌Osaka, Japan; Versiti Blood Research Institute, UNITED STATES OF AMERICA

## Abstract

Aging is strongly associated with an increased risk of morbidity and mortality from multiple diseases, including thromboembolic disorders. Endothelial dysfunction is considered a key contributor to age-related thrombus formation, although its underlying mechanisms remain incompletely understood. Cellular senescence is a fundamental driver of aging; however, the distinct functional roles of replicative (RS) and stress-induced premature senescence (SIPS) in cellular functions remain unclear. This study, investigated the effects of endothelial cell (EC) senescence on blood coagulation, and demonstrated that RS and SIPS differentially regulate endothelial anticoagulation capacity. Plasma coagulation capacity, assessed using calibrated automated thrombogram, was unexpectedly reduced in the presence of RS-ECs compared with that in the presence of young control cells, whereas SIPS-EC showed no such effect. RNA sequencing analysis revealed distinct global transcriptional and coagulation pathway-related alterations between RS- and SIPS-ECs. Despite enhanced anticoagulation capacity in RS-ECs *in vitro*, thrombus formation was exacerbated in naturally aged mice *in vivo*. The contribution of SIPS-ECs to thrombus formation was further evaluated *in vivo* using EC-specific SIPS mouse models. EC-specific SIPS mice exhibited aggravated venous thrombus formation, with thrombus histological features resembling those observed in naturally aged mice. Gene expression profiles related to blood coagulation were also largely similar between ECs isolated from naturally aged and EC-specific SIPS mice. These findings demonstrate distinct contributions of endothelial RS and SIPS to blood coagulation and suggest that SIPS, rather than RS, may represent the predominant form of endothelial senescence during *in vivo* aging with respect to age-related dysregulation of blood coagulation.

## Introduction

Population aging represents a major global health challenge because advanced age significantly contributes to morbidity and mortality across multiple diseases [1,2]. Cardiovascular diseases remain the leading cause of death worldwide. According to the World Health Organization, approximately 18 million deaths in 2019 were attributed to cardiovascular diseases, accounting for 32% of all global mortality. Among these, 85% resulted from heart attacks and strokes, primarily caused by a thrombus formation within blood vessels. Blood coagulation is tightly regulated by procoagulant and anticoagulation pathways, and disruption of this balance leads to thrombotic or bleeding disorders [3]. Advanced age is strongly associated with an increased incidence of both conditions [4,5].

Endothelial cells (ECs), which line the inner surface of entire blood vessels, are in direct contact with circulating blood and play essential roles in maintaining blood fluidity through regulation of coagulation, platelet activity, and fibrinolysis [6]. Healthy ECs maintain anticoagulant and antiplatelet properties, whereas EC dysfunction promotes fibrin formation, platelet adhesion, and thrombus formation [7]. Vascular aging is increasingly recognized as a major contributor to endothelial dysfunction and is closely linked to elevated cardiovascular risk in the elder populations [8,9].

Cellular senescence, defined as an irreversible growth arrest, plays a central role in biological aging [10,11]. Endothelial senescence contributes substantially to vascular aging and age-related vascular diseases [12]. Previous studies have demonstrated that senescent ECs are involved in the pathogenesis of multiple conditions, including diabetes, atherosclerosis, pulmonary hypertension, cancer hematogenous metastasis, and COVID-19 [13–17]. However, the specific role of endothelial senescence in blood coagulation and thrombus formation remains poorly understood. Cellular senescence is broadly categorized into replicative senescence (RS) and stress-induced premature senescence (SIPS) [18–20]. RS is primarily driven by telomere dysfunction resulting from repeated cell division, whereas SIPS occurs independently of telomere length and is induced by oxidative stress, mitochondria deterioration, and oncogenic signaling [20–22]. Although RS and SIPS share common phenotypic features, including senescence-associated secretory phenotype (SASP), potential functional differences remain insufficiently characterized [20]. Both forms involve DNA damage response pathways, which play critical roles in senescence induction and SASP regulators [23]. Consequently, RS and SIPS are often difficult to distinguish, and mechanistic differences in their contributions to aging remain unclear. This study investigated the roles of RS and SIPS in endothelial function, particular focus on anticoagulation capacity. In addition, the contribution of SIPS-ECs to venous thrombus formation was examined using genetically modified mice wherein SIPS was specifically induced in ECs [13].

## Materials and methods

### Reagents

Anti-human myeloperoxidase (MPO) antibody was obtained from R&D Systems (#AF3667). Antibodies against myc and von Willebrand factor were purchased from MBL life science (#M192-3) and Abcam (#ab9378), respectively. Alexa Fluor 488 donkey antirabbit (#A21206) and Alexa Fluor 594 donkey antimouse (#A21203) secondary antibodies were obtained from Invitrogen.

Human umbilical vein endothelial cells (HUVECs) were obtained from Lifeline Cell Technology. Thrombinoscope equipment and reagents for calibrated automated thrombogram (CAT), including FluCa Kit (#86322), calibrator (#86192), and platelet-rich plasma (PRP) reagent (#86196), were obtained from Stago Group.

### Cell culture

HUVECs were cultured in HuMediaEG2 medium (Kurabo #KE-2350S) and regularly passaged at a 1:4 ratio upon reaching subconfluence. Cells at passage 3–5 were used as young controls.

RS was induced through extended passages until 17–20. We previously reported that RS-ECs highly express the SASP factors [13]. SIPS was induced by lentiviral overexpress of dominant-negative form of telomere repeat-binding factor 2 (TRF2). Enhanced SASP factor expression in these SIPS-ECs was confirmed (S1A Fig).

### RNA sample preparation, RNA sequencing, and data processing

HUVECs at passages 3–5 were designated as young controls, whereas cells at passages 17–20 were classified as RS. In addition, SIPS was induced through overexpression of dominant-negative mutant of the telomere-binding protein TRF2 (TRF2ΔBΔM).

Total RNA was extracted using TRIzol (Invitrogen #15596018) and purified using the Direct-zol RNA MiniPrep kit (Zymo Research #R2052). RNA quantity and integrity were assessed using NanoDrop spectrophotometry and Agilent 4200 TapeStation analysis. RNA libraries were prepared using the Illumina TruSeq RNA Exome kit, followed by paired-end sequencing (2 × 100 bp) on the Illumina NovaSeq 6000 platform.

Raw FASTQ files were quality-checked using FastQC (v0.12.1), and adapter trimming was performed with Trim Galore (v0.6.10). Reads were aligned to the Homo sapiens reference genome (GRCh38, GENCODE release 44) using STAR (v2.7.11a) in two-pass mode. Gene-level read counts were quantified using feature Counts (v2.0.6) with exon features in strand-specific, paired-end mode, including chimeric read handling. Count matrices were analyzed in R (v4.4.3) using DESeq2 (v1.46.0). Size factors were estimated using the median-of-ratios method, followed by variance-stabilizing transformation. Principal component analysis (PCA) was performed on normalized data to assess global transcriptional variation. Differential expression analysis was conducted using the Wald test with log₂ fold-change shrinkage implemented through the ashr method. Genes with adjusted p value < 0.05 and |log₂FC| > 1 were considered significantly differentially expressed and were subsequently subjected to pathway enrichment analysis using the ReactomePA package (v1.50.0) based on the Reactome database.

### Animals

All animal procedures were approved by the Ethics Review Committee for Animal Experimentation of the Kyoto Prefectural University of Medicine (M2022-117, M2023–105, and M2024-89) and conducted in accordance with with the Animal Research: Reporting of In Vivo Experiments guidelines and relevant regulations.

Mice were housed in designated cages of sufficient size (1–3 mice in one cage) in an animal facility where the temperature and humidity were regulated at 23℃ and 60%, respectively. Mice were given ad libitum access to water and food under a 12-h light/12-h dark cycle.

Endothelial-specific SIPS mice overexpressing the dominant negative form of TRF2 in ECs (C57/BL6J background) were used as previously described [13,15] with littermate wild-type (WT) mice serving as controls.

### CAT assay

Thrombin generation in human plasma was assessed using the CAT system (Stago Group). Young, RS, or SIPS-ECs were seeded onto assay plates 24 h before measurement. Platelet-rich plasma (80 µL) was added to each well, followed by PRP reagent calibrator (20 µL). FluCa reagent (20 µL) was automatically dispensed, and thrombogram generation was initiated. Coagulation parameters were calculated from resulting thrombograms (S1 Fig).

### Inferior vena cava ligation model

Inferior vena cava (IVC) ligation models were established using young (8−12 weeks) or naturally aged (90−100 weeks) WT mice, as well as dominant-negative form of telomeric repeat-binding factor 2 (TRF2DN)-Tg or littermate WT mice controls aged 20−30 weeks, as previously described [24]. Each experimental group included 6−10 male mice. All animal welfare considerations were implemented, including measures to minimize suffering and distress, appropriate use of analgesics or anesthetics, and standardized housing conditions. Research personnel were trained in animal care and handling by experienced senior investigators.

The experimental duration was 1 day. On day 0, mice were anesthetized with 2% isoflurane and underwent surgical permanent IVC ligation. A midline lower abdominal incision was performed, and the aorta was carefully separated from the IVC. The right branch of the IVC was ligated, followed by complete ligation of the main IVC trunk distal to the bifurcation of the left renal vein (S2A Fig). Animal health and behavior were monitored 2 h following surgery. Humane endpoints included bleeding from natural orifices, abnormal posture, swelling or edema, dyspnea, tachypnea, or significant deterioration in body condition. No mice was sacrificed during the study or reached predefined humane endpoints. On day 1, mice were anesthetized with 2% isoflurane and euthanized by cervical dislocation. IVC thrombi were subsequently collected, measured for length and weight (S2B Fig), and then fixed in 4% paraformaldehyde for histological analysis.

### Immunohistochemistry

Neutrophil infiltration was evaluated by immunohistochemical staining for MPO using frozen thrombus sections. Following blocking with 10% normal donkey serum, sections were incubated overnight at 4℃ with anti-MPO antibody (1:200, 5 µg/mL), normal goat IgG (Wako #580–98321, 1:200, 5 µg/mL) served as the negative control. After phosphate buffered saline (PBS) washing, sections were incubated with fluorescence-labeled secondary antibodies (1:500), mounted with DAPI-containing antifade medium (Vector Laboratories #H-1200), and analyzed using fluorescence microscope (Keyence).

TRF2DN expression in endothelial-specific transgenic mice was assessed by myc immunostaining of whole-lung sections obtained from 11- to 12-week-old WT and EC-specific TRF2DN transgenic mice. Sections were incubated with antibodies against myc (1:100, 10 µg/mL) and von Willebrand factor (1:200, 50 µg/mL), followed by Alexa Fluor 488 donkey antirabbit (1:300) and Alexa Fluor 594 donkey antimouse secondary antibodies (1:300). After PBS washing, sections were mounted with DAPI-containing antifade medium (Vector Laboratories #H-1200) and imaged using Keyence BZ-X800 fluorescence microscopy.

### Quantitative RT-PCR

Quantification of target gene mRNA expression was performed as previously described [13]. Total RNA was isolated from HUVECs using TRIzol (Invitrogen) and purified with the Direct-zol RNA MiniPrep kit (Zymo Research). Complementary DNA was synthesized using PrimeScript RT Master Mix (TaKaRa), followed by quantitative PCR using the CFX384 system (BioRad).

Gene expression levels were quantified using the delta-delta Ct method and normalized to β-actin or GAPDH expression.

### Isolation of ECs from mouse lungs

ECs were isolated from the lungs of aged and EC-specific SIPS mice using a magnetic-activated cell sorting (MACS) system (Miltenyi Biotec #130-095-927). Lung tissues were gently dissociated using the Lung Dissociation kit (Miltenyi Biotec #130-095-927) and gentle MACS Dissociator (Miltenyi Biotec #130-093-235) according to the manufacturer’s instructions. ECs were subsequently isolated using CD146 (LSEC) Microbeads (Miltenyi Biotec #130-092-007).

### Statistical analysis

Statistical analyses were performed using the GraphPad Prism 10. Data are presented as mean ± SEM. Normality was assessed using the Shapiro–Wilk test. Comparisons between two groups were performed using two-tailed unpaired Student’s t-test, whereas comparisons between multiple groups were analyzed using one-way ANOVA with Fisher’s least significant difference post hoc test.

## Results

### Replicative senescent ECs reduced thrombin generation in human plasma

**Fig 1:**
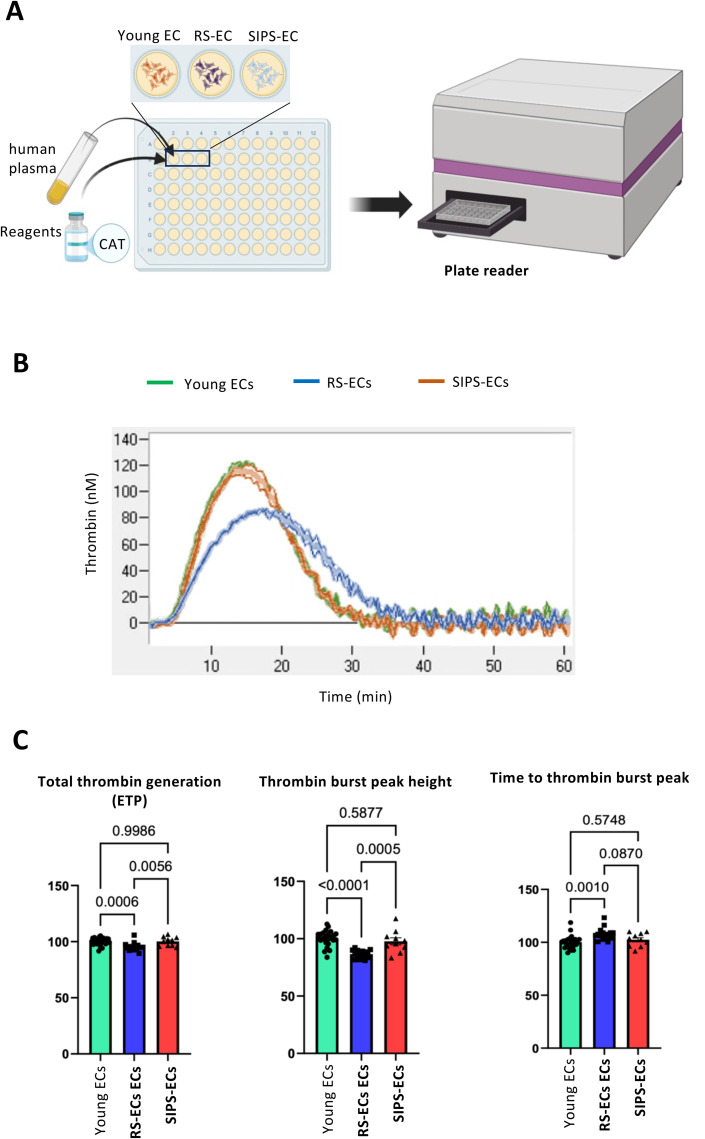
RS enhances the anticoagulation capacity in ECs. (A) Schematic overview of experimental design. (B) Blood coagulability was assessed by CAT assay using human plasma. Representative data using duplicate samples are shown. (C) Quantification of endogenous thrombin generation, (ETP) thrombin burst peak time, and time to thrombin burst peak (n = 25 for young; n = 15 for RS; n = 10 for SIPS). The presence of RS-EC reduced ETP and thrombin burst peak height, while prolonging the time to thrombin burst peak compared with young control ECs. Data are presented as the mean ± SEM, with corresponding P-values shown in each graph.

To investigate the role of EC senescence in thromboembolic diseases, the effects of replicative senescent endothelial cells (RS-ECs) and stress-induced premature senescent ECs (SIPS-ECs) on blood coagulation were evaluated. Young ECs (passages 4–5) served as controls. Blood coagulation was quantitatively assessed using human PRP through the CAT assay (Fig 1A). Endogenous thrombin potential (ETP), peak height of the thrombin burst, and the time to the peak were analyzed (S1B Fig). Unexpectedly, RS-ECs significantly reduced ETP, decreased peak thrombin generation, and prolonged time to peak compared with young control ECs (Fig 1B and 1C). In contrast, SIPS-ECs did not significantly alter these anticoagulation parameters. These findings suggest that RS, but not SIPS, enhances endothelial anticoagulation capacity.

### Transcriptional alteration induced by cellular senescence was substantially different between RS and SIPS

**Fig 2:**
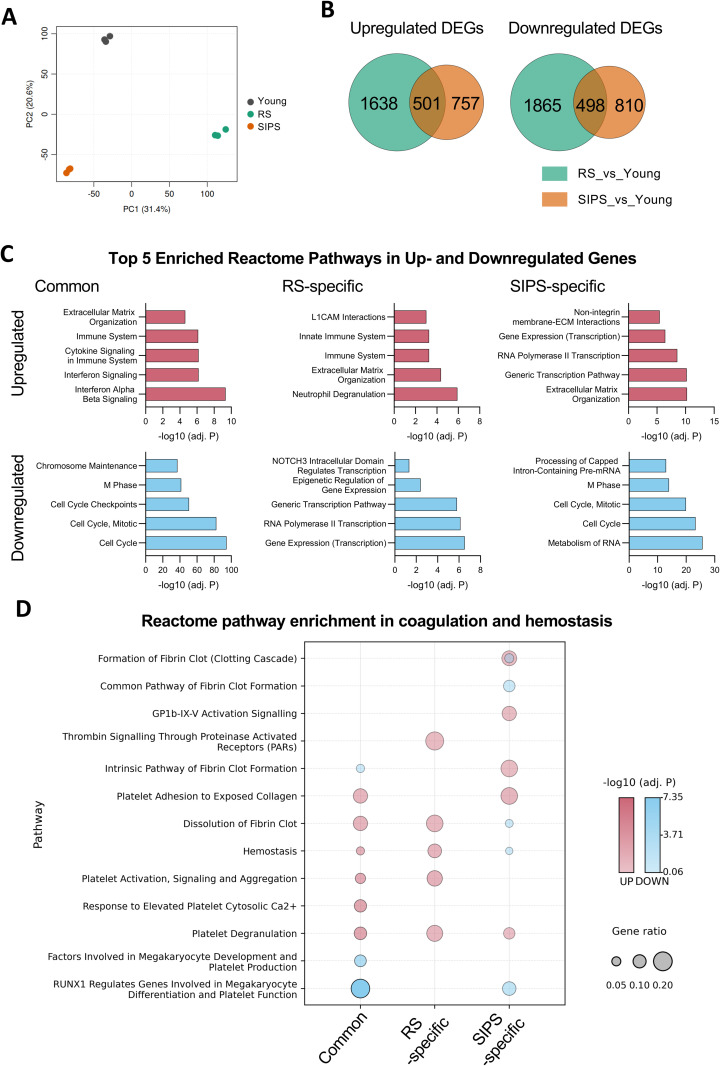
Transcriptomic characterization of young, replicative senescent, and premature senescent ECs. (A) Principal component analysis (PCA) of RNA-seq data from young (gray), replicative senescent (green), and premature senescent (orange) ECs (n = 3 per group). (B) Proportional Venn diagrams showing overlap of differentially expressed genes (DEGs) in replicative (green) and premature (orange) senescent ECs versus young controls. Left, upregulated (1638/501/757), right, downregulated (1865/498/810) genes, presented as replicative-specific / common / premature-specific. (C) Top five enriched Reactome pathways identified from common, replicative-specific, and premature-specific DEGs. Bar graphs show upregulated (red) and downregulated (blue) pathways for each group. (D) Reactome enrichment analysis of coagulation and hemostasis pathways. *Dot plot* displays terms derived from common, replicative-specific, and premature-specific DEGs relative to young controls. *Dot color* indicates direction (red up, blue down), opacity represents −log10 (adjusted *P* value), and dot size reflects gene ratio; right corresponding scales.

Transcriptomic profiles of young, RS-, and SIPS-ECs were analyzed using RNA sequencing. PCA demonstrated clear segregation among the three groups, indicating distinct global transcriptional patterns (Fig 2A). Differential expression analysis identified 2,139 upregulated and 2,363 downregulated genes in the RS group relative to the young group, and 1,258 upregulated and 1,308 downregulated genes in the SIPS group (Fig 2B). Among these, 501 genes were commonly upregulated and 498 genes were commonly downregulated in both senescent groups compared with the young controls. Reactome pathway enrichment analysis of shared differentially expressed genes (DEGs) revealed common enrichment of inflammation-related pathways and suppression of cell-cycle-related pathways across senescence models (Fig 2C).

Notably, each senescence model exhibited distinct transcriptional alterations. In the RS group, upregulated pathways were primarily related to immune responses, while pathways involved in transcriptional regulation were downregulated. In contrast, the SIPS group showed enhanced transcriptional activity, accompanied by significant downregulation of cell-cycle-related pathways.

To further assess differences in anticoagulant properties between RS- and SIPS-ECs, coagulation- and hemostasis-related pathways were analyzed. Commonly upregulated DEGs in both senescent groups were strongly enriched in platelet activation and early hemostasis pathways, whereas commonly downregulated DEGs were markedly depleted in megakaryocyte- and platelet-associated programs (Fig 2D). Distinct coagulation-related signatures were observed between senescence models. RS-specific DEGs showed moderate enrichment in platelet activation and fibrinolysis pathways, whereas SIPS-specific DEGs were preferentially associated with platelet adhesion and early coagulation components. Thus, both senescence processes engaged coagulation biology through distinct molecular mechanisms: RS favored platelet activation and fibrinolysis, whereas SIPS favored platelet adhesion and intrinsic coagulation. These findings highlight the differential remodeling of platelet and coagulation networks across endothelial aging states. In addition, direct transcriptomic comparison between RS- and SIPS-ECs revealed substantial differences despite shared senescent phenotypes (S2 Figs). Reactome pathway enrichment analysis demonstrated enrichment of SASP, DNA modification, and cell cycle checkpoint pathways in RS-ECs, whereas extracellular-related pathways were more prominent in SIPS-ECs (S2B Fig). Identification of genes uniquely associated with RS or SIPS remains valuable, although current data were insufficient for definitive characterization.

### Thrombus formation was exacerbated in aged and EC-specific SIPS mice

**Fig 3:**
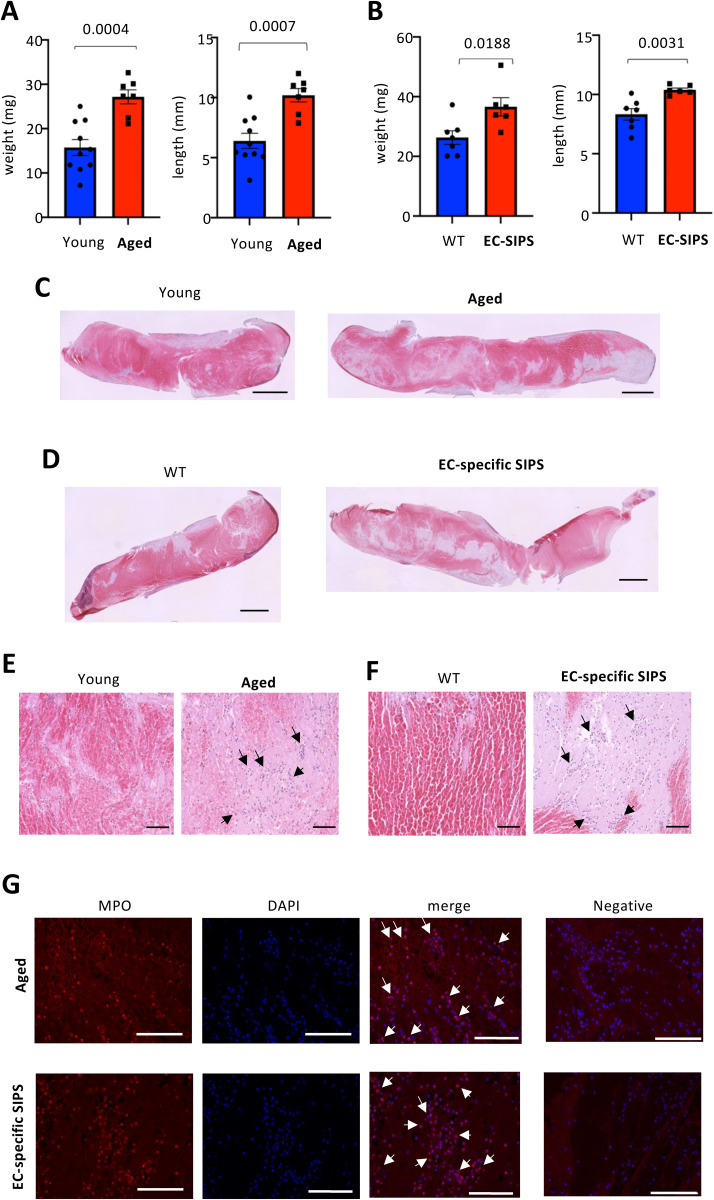
Aging exacerbates venous thrombus formation. (A) IVC ligation was performed in young and aged WT mice; thrombus weight and length quantified (n = 10 for young; n = 7 for aged). Data presented as the mean ± SEM; P-values are shown in each graph. (B) IVC ligation was performed in littermate (WT) and EC-specific SIPS (EC-SIPS) mice; thrombus weight and length quantified (n = 6 per group). Data presented as the mean ± SEM; P-values are indicated. (C) H&E-stained thrombus sections from young and aged WT mice. Bars: 1 mm. (D) H&E-stained thrombus sections from WT and EC-specific SIPS mice. Bars: 1 mm. (E) Higher magnification of thrombus sections from young and aged WT mice; arrows indicate infiltrating cells. Bars: 100 µm. (F) Higher magnification of thrombus sections from littermates and EC-specific SIPS mice; arrows indicate infiltrating cells. Bars: 100 µm. (G) MPO immunohistochemistry of thrombus sections isolated from aged WT and EC-specific SIPS mice; normal goat IgG used as negative control. Arrows indicate MPO-positive neutrophils. Bars: 100 µm. Data presented as the mean ± SEM; P-values are indicated.

A venous thrombus model was established by IVC ligation (S3B Fig). Thrombi were collected 24 h postligation and quantified by weight and length (S3C Fig). First, we investigated whether aging affects thrombus formation using young and aged WT mice. Despite the enhanced anticoagulation capacity in RS-ECs *in vitro*, the weight and length of the thrombi were considerably increased in aged mice than in young mice, indicating the enhanced blood coagulation in aged mice (Fig 3A). EC-SIPS mice were generated via overexpression of TRF2DN [13–17]. The expression of TRF2DN in ECs was confirmed by immunohistochemistry in these mice (S3A Fig). Application of the same IVC ligation model revealed increased thrombus formation in EC-specific SIPS mice as well (Fig 3B).

We then analyzed the histological features of the thrombus. Marked cellular infiltration in clusters was detected within the thrombi formed in both aged and EC-specific SIPS mice but not in the thrombi formed in young and littermate WT mice (Fig 3C–F). Quantification confirmed increased cellular infiltration in both aged and EC-specific SIPS thrombi (S3D Fig).

Immune activation contributed to thrombosis through leukocyte-derived proinflammatory and procoagulant factors [25]. Neutrophils represent the predominant leukocyte population in venous thrombi [26]. Immunohistochemical staining for MPO identified infiltrating cells as predominantly MPO-positive neutrophils in aged and EC-specific SIPS thrombi (Fig 3G). These data indicate dysregulated leukocyte recruitment and innate immune activation in aging-associated thrombosis. Furthermore, endothelial SIPS, rather than RS, appears to be a primary driver of coagulation dysregulation with aging.

### Expressional changes in coagulation- and fibrinolysis-related genes were similar in ECs from aged and EC-specific SIPS mice

**Fig 4:**
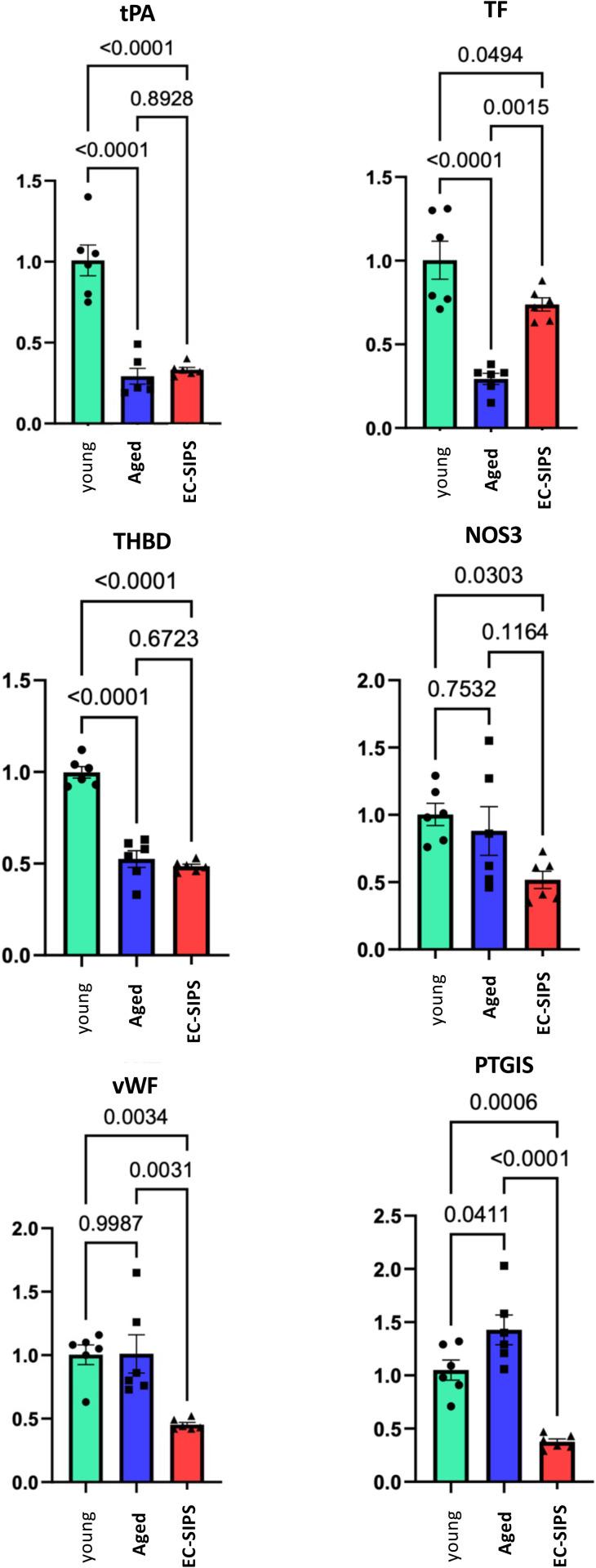
Expressional changes in genes related to coagulation and fibrinolysis in ECs isolated from aged and EC-specific SIPS mice. ECs were isolated from the lungs of young WT, aged WT, and EC-specific SIPS mice. Quantitative PCR analysis of tissue plasmin activator (tPA), tissue factor (TF), thrombomodulin (THBD), NO synthase 3 (NOS3), von Willebrand factor (vWF), and prostaglandin I2 synthase (PTGIS) was performed (n = 6 per group). Data are presented as the mean ± SEM. P-values are indicated.

ECs were isolated from lungs of young WT, aged WT, and EC-specific SIPS mice. Gene expression related to coagulation and fibrinolysis was assessed by quantitative PCR. Expression of tissue plasmin activator, tissue factor, and thrombomodulin was comparably reduced in ECs from aged and EC-specific SIPS mice relative to young controls (Fig 4). In contrast, NO synthase 3, von Willebrand factor, and prostaglandin I2 synthase were decreased only in ECs from EC-specific SIPS mice (Fig 4). These findings indicate overlapping alterations in coagulation- and fibrinolysis-related pathways and support a central role of SIPS in endothelial aging. In addition, we assessed the expressional changes of these genes in young, RS-, and SIPS-HUVECs using the RNA-seq dataset. In contrast to mouse lung ECs, almost no change was detected between young and SIPS-HUVECs (S4 Fig). Variability may reflect differences in senescence extent or EC subtype. Of note, expression of tissue plasmin activator, thrombomodulin, and NO synthase 3 was significantly increased in RS-HUVECs relative to young cells (S4 Fig), consistent with enhanced anticoagulation capacity observed in CAT assays.

## Discussion

Ischemic cardiovascular diseases, including myocardial and cerebral infarction, represent the leading cause of mortality worldwide and show high prevalence in elderly populations. Increased blood stasis due to immobility, elevated coagulation factors, and enhanced platelet reactivity contribute to increased thromboembolic risk with aging [27,28]. EC dysfunction also contributes to age-related thrombogenicity through regulation of blood fluidity and homeostasis [6]. However, the contribution of cellular senescence in ECs to coagulation remains unclear, and differential role of RS and SIPS in EC function remain insufficiently defined.

RS results from repeated cell division and telomere shortening, leading to growth arrest [29,30]. Telomere length correlates with age and lifespan [31], although associations with replication capacity remain inconsistent [32,33]. In contrast, SIPS occurs independently of telomere length and is induced by stressors such as oncogene activation and oxidative stress. RS and SIPS share phenotypic features, including cell cycle arrest, increased senescence-associated *β*-galactosidase activity, DNA damage response, and SASP. Despite shared features, distinct functional roles remain unclear. Quantitative assessment of RS versus SIPS population remains challenging and likely varies by tissue, age, and health status. Previous report showed that the proteomics analysis identified 24 and 10 altered proteins in RS and SIPS cells, respectively, with minimal overlap [34]. In addition, vascular smooth muscle cells with SIPS lack mineralization observed in RS-cells [35].

The present study evaluated effects of RS and SIPS on endothelial function, focusing on anticoagulation capacity. RS-ECs demonstrated increased anticoagulation capacity *in vitro* based on CAT assays. In contrast, aged mice exhibited increased thrombus formation relative to young controls. EC-specific SIPS mice also showed enhanced thrombus formation, accompanied by increased clustered neutrophil infiltration. Study limitations include reliance on mouse models and CAT assays constraints. TRF2DN-induced SIPS may not fully recapitulate stress-induced senescence during aging, and CAT assays do not capture the full coagulation process. Nevertheless, findings support SIPS as a predominant senescence mechanism in aging ECs.

Leukocytes, particularly neutrophils, contribute significantly to thrombus formation [25]. Neutrophils dominate leukocyte populations within thrombi and promote thrombosis through neutrophil extracellular traps [36,37]. Reduced cellular infiltration in thrombi from young mice compared with aged mice suggests enhanced neutrophil recruitment during aging. Indeed, E-selectin and VCAM-1, which are adhesion molecules that play an important role in the leukocytes recruitment, were markedly increased in SIPS-HUVECs *in vitro* (S5A Fig). Similar results were detected when compared between lung ECs isolated from young WT ‌‌and EC-specific SIPS mice (S5B Fig).

RS and SIPS exert distinct effects on endothelial function, particularly anticoagulation capacity. Endothelial SIPS likely contributes to age-related thromboembolic risk. SIPS-ECs provide a more relevant model than RS-ECs for studying aging-associated endothelial dysfunction. Differential functional effects of RS and SIPS necessitate careful selection of senescence models in aging research.

## Supporting information

S1 Fig Thrombogram parameters. (A) Expression of the SASP factors in young and SIPS-HUVECs was quantitatively analyzed (n = 5 for young; n = 7 for SIPS). Data presented as the mean ± SEM; P-values are indicated. (B) Representative curves from the CAT assay.
(PDF)

S2 Fig Transcriptomics comparison between RS- and SIPS-HUVECs. (A) Volcano plots of differentially expressed genes (DEGs) between RS- and SIPS-HUVECs were shown. (B) The top 5 enriched reactome pathways enriched in either RS- or SIPS-ECs.
(PDF)

S3 Fig Generation of the IVC ligation model in mice. (A) Detection of myc-tagged TRF2DN in mouse lung endothelial cells by immunohistochemistry. Colocalization was observed with vWF-positive ECs (arrows). Bars: 50 µm. (B) Schematic diagram of IVC ligation. (C) Representative image of thrombus formed in the IVC. (D) The number of cells infiltrated in thrombi was quantitatively analyzed (n = 4 for young, aged, and WT; n = 5 for EC-specific SIPS). Data presented as mean ± SEM; P-values are indicated.
(PDF)

S4 Fig Expression of genes related to blood coagulation. Expression of coagulation-related genes in young, RS-, and SIPS-HUVECs was assessed using the RNA-seq dataset. Data presented as mean ± SEM; P-values are indicated
(PDF)

S5 Fig Quantitative PCR analysis of adhesion molecules. (A) Expression of E-selectin (SELE) and vascular cell adhesion molecule-1 (VCAM-1) in young control and SIPS-HUVECs (n = 6 per group). (B) Expression of SELE and VCAM-1 in ECs isolated from the lungs of young and EC-specific SIPS mice (n = 6 per group). Data presented as mean ± SEM; P-values are indicated.
(PDF)
