# Supplementary figures and images for: Replicative and stress-induced premature senescence distinctively affect the endothelial anticoagulation capacity
Source: PLoS One. 2026 Jun 9;21(6):e0351140. doi: 10.1371/journal.pone.0351140 (PMC13249167; doi:10.1371/journal.pone.0351140)

**S1 Fig.**

**A**

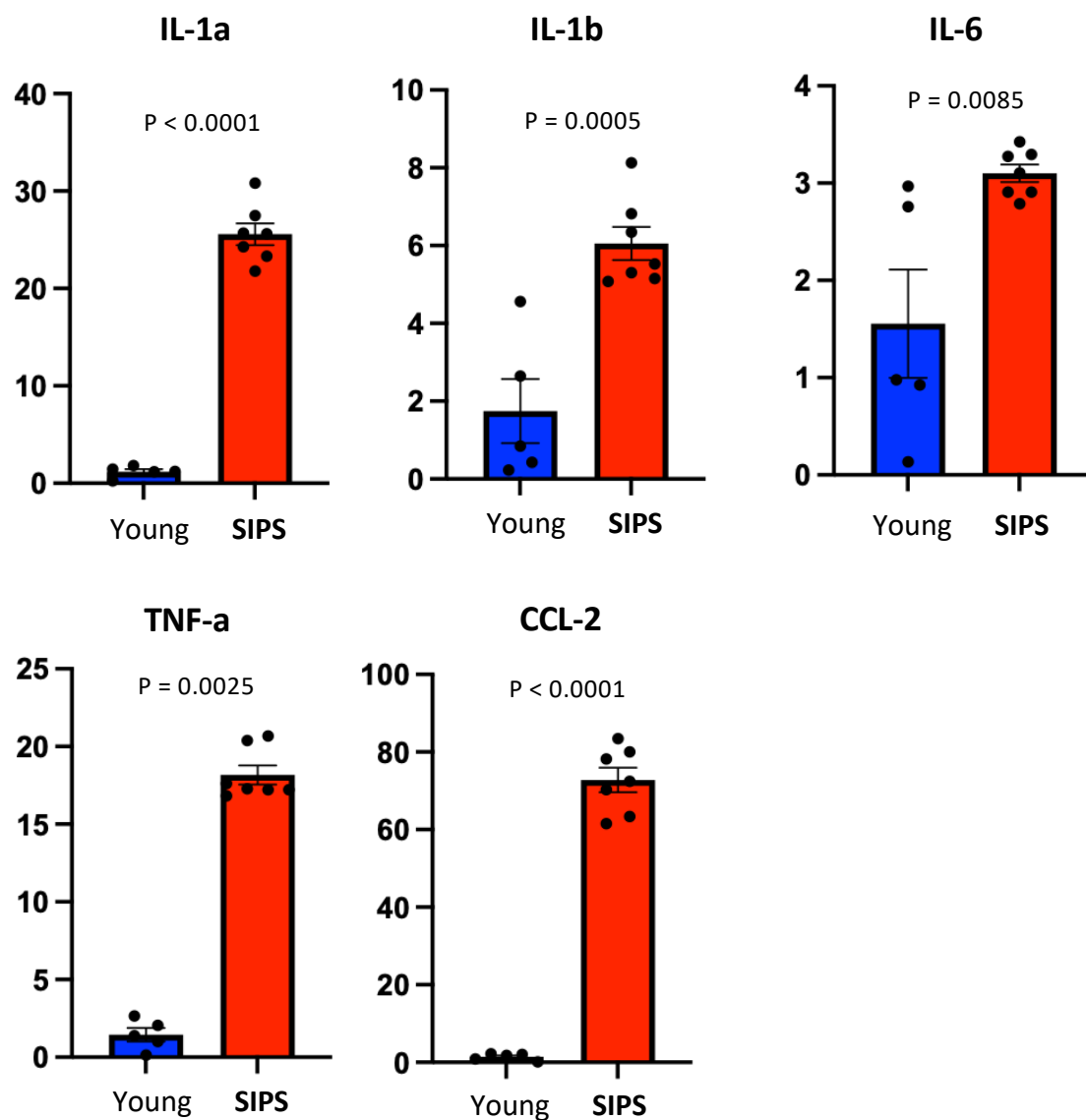

**B**

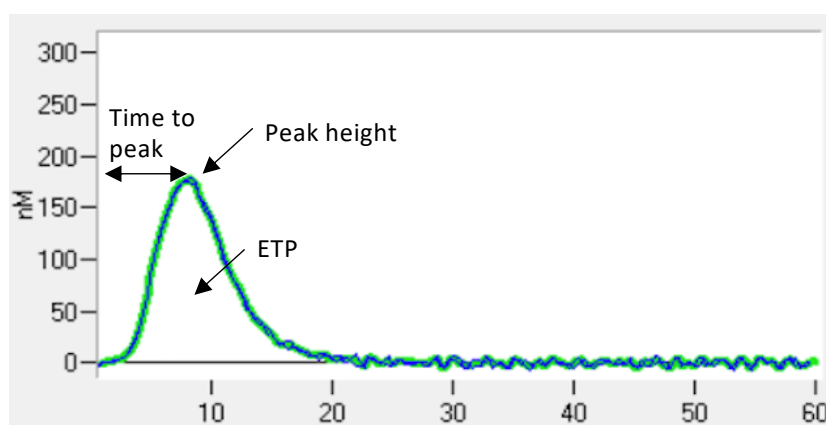

Supplement: S1 Fig — (A) Expression of the SASP factors in young and SIPS-HUVECs was quantitatively analyzed (n = 5 for young; n = 7 for SIPS). Data presented as the mean ± SEM; P-values are indicated. (B) Representative curves from the CAT assay. (PDF) [file pone.0351140.s001.pdf]

S3 Fig.

A

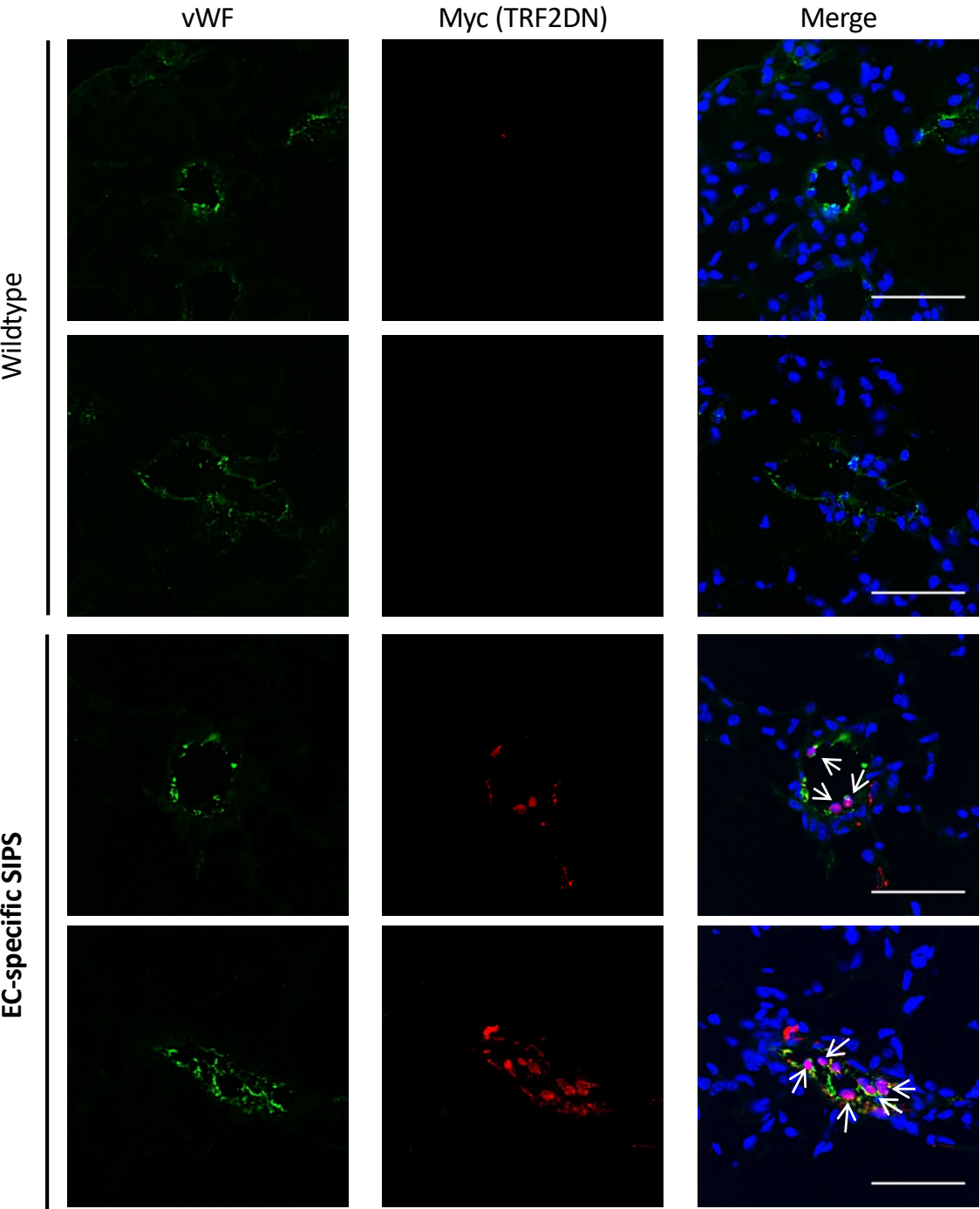

S3 Fig.

B

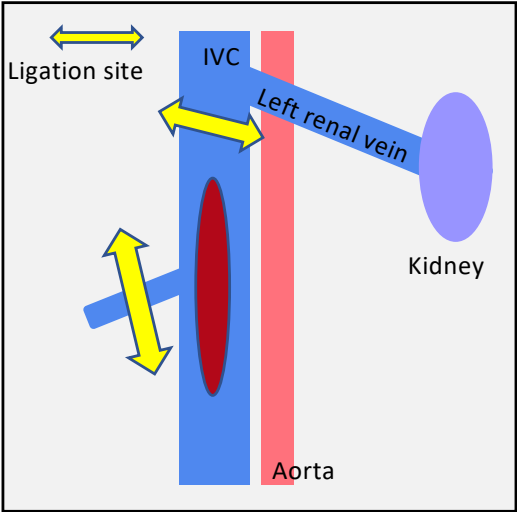

C

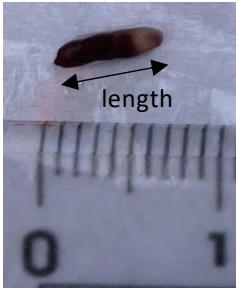

D

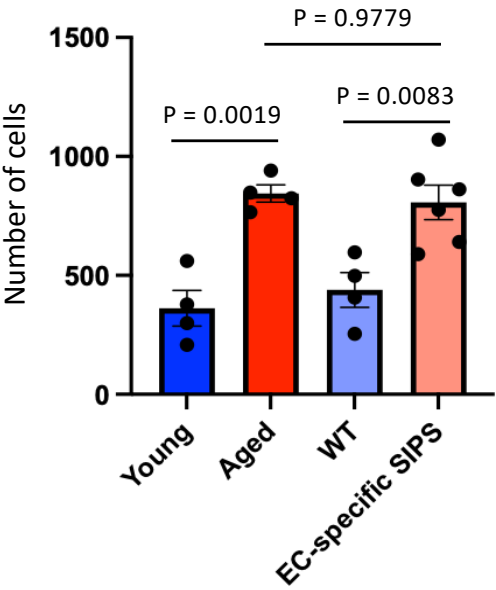

Supplement: S3 Fig — (A) Detection of myc-tagged TRF2DN in mouse lung endothelial cells by immunohistochemistry. Colocalization was observed with vWF-positive ECs (arrows). Bars: 50 µm. (B) Schematic diagram of IVC ligation. (C) Representative image of thrombus formed in the IVC. (D) The number of cells infiltrated in thrombi was quantitatively analyzed (n = 4 for young, aged, and WT; n = 5 for EC-specific SIPS). Data presented as mean ± SEM; P-values are indicated. (PDF) [file pone.0351140.s003.pdf]

S4 Fig.

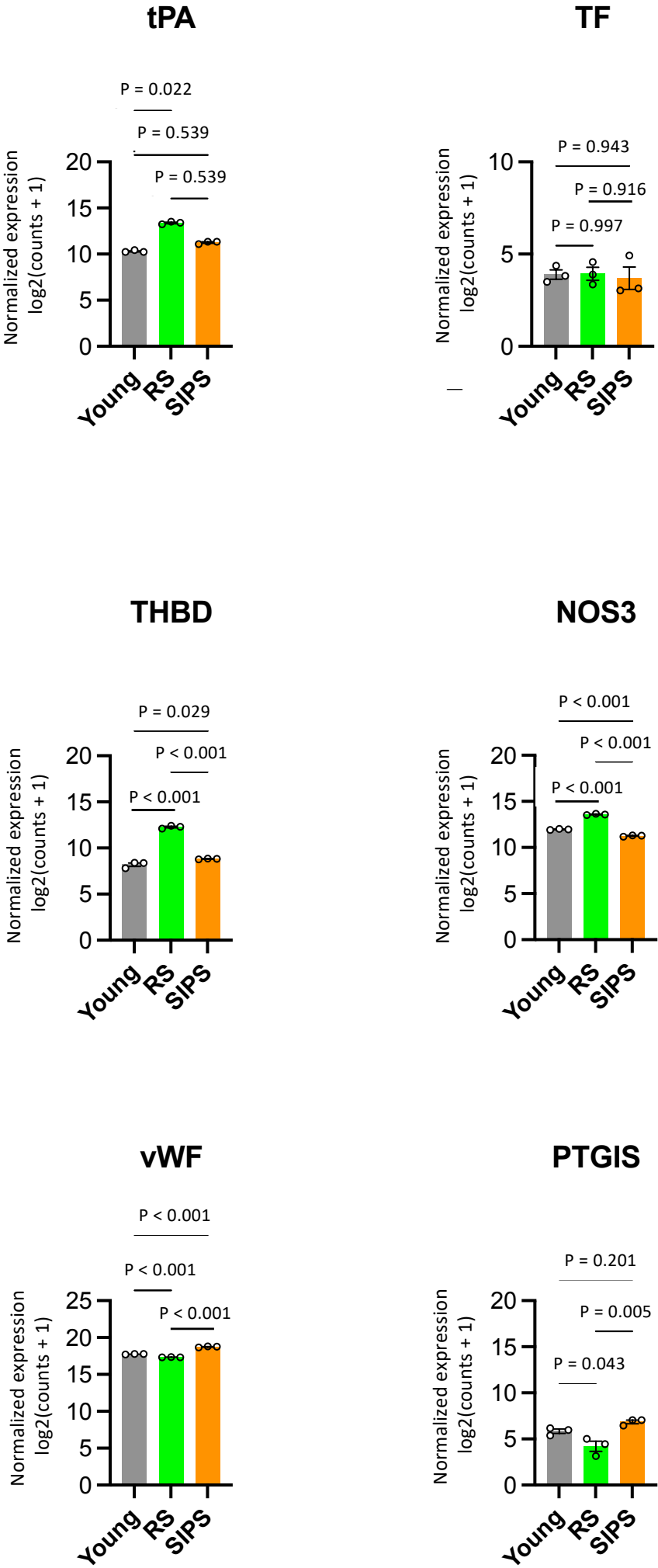

Supplement: S4 Fig — Expression of coagulation-related genes in young, RS-, and SIPS-HUVECs was assessed using the RNA-seq dataset. Data presented as mean ± SEM; P-values are indicated (PDF) [file pone.0351140.s004.pdf]

**S5 Fig.**

**A**

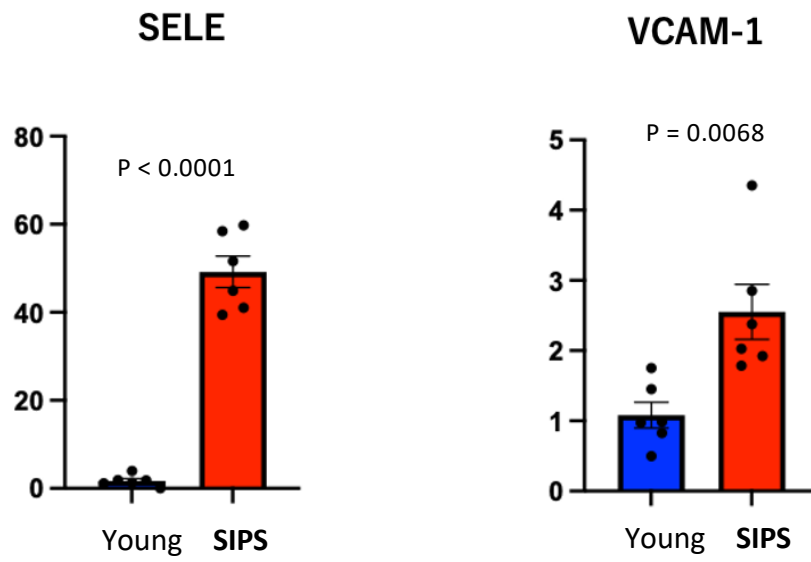

**B**

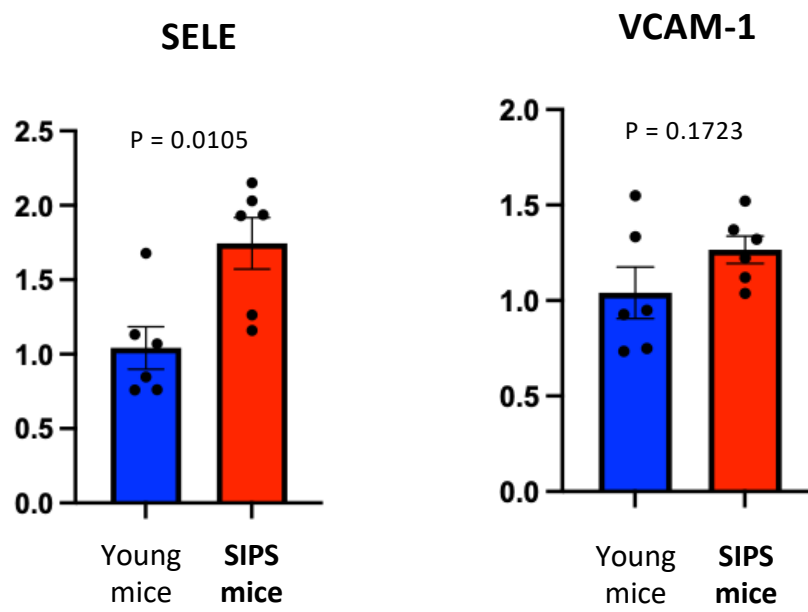

Supplement: S5 Fig — (A) Expression of E-selectin (SELE) and vascular cell adhesion molecule-1 (VCAM-1) in young control and SIPS-HUVECs (n = 6 per group). (B) Expression of SELE and VCAM-1 in ECs isolated from the lungs of young and EC-specific SIPS mice (n = 6 per group). Data presented as mean ± SEM; P-values are indicated. (PDF) [file pone.0351140.s005.pdf]
