# Supplementary material for: Replicative and stress-induced premature senescence distinctively affect the endothelial anticoagulation capacity
Source: PLoS One. 2026 Jun 9;21(6):e0351140. doi: 10.1371/journal.pone.0351140 (PMC13249167; doi:10.1371/journal.pone.0351140)

S2 Fig.

A

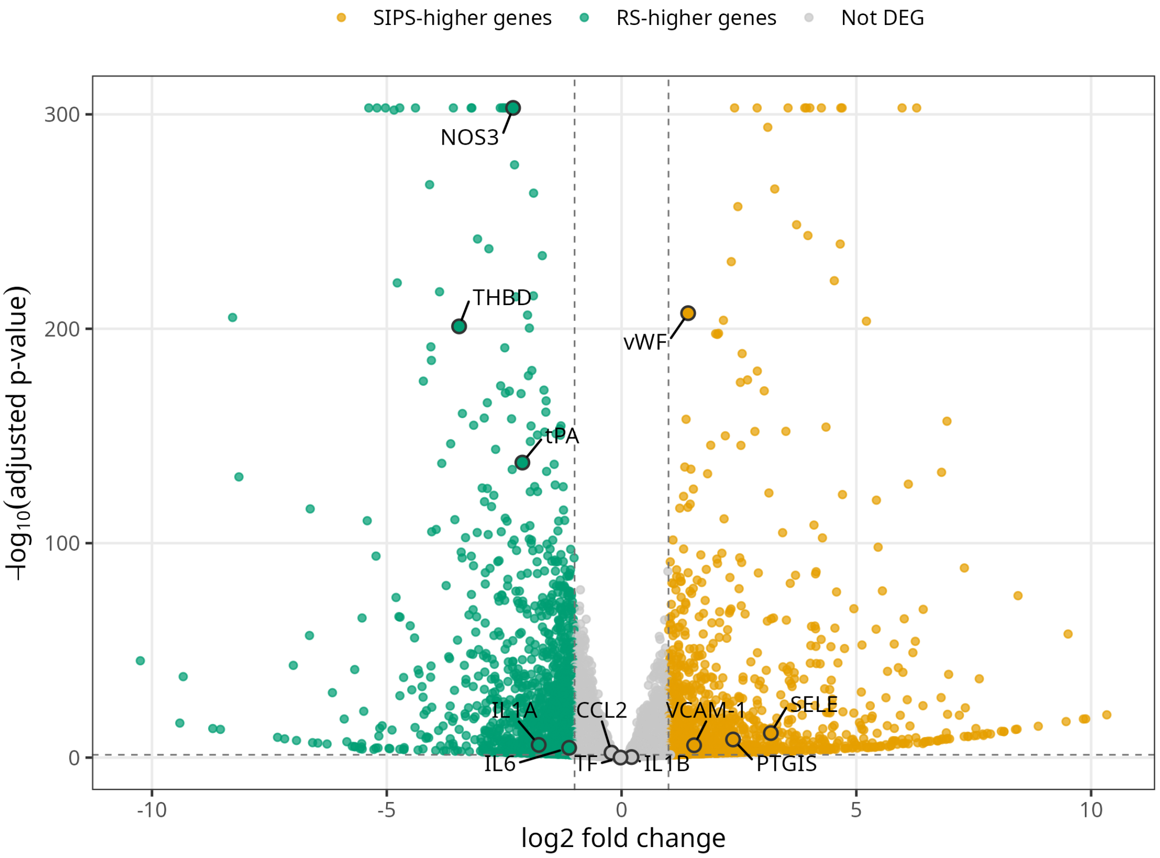

B

Top 5 Enriched Reactome Pathways

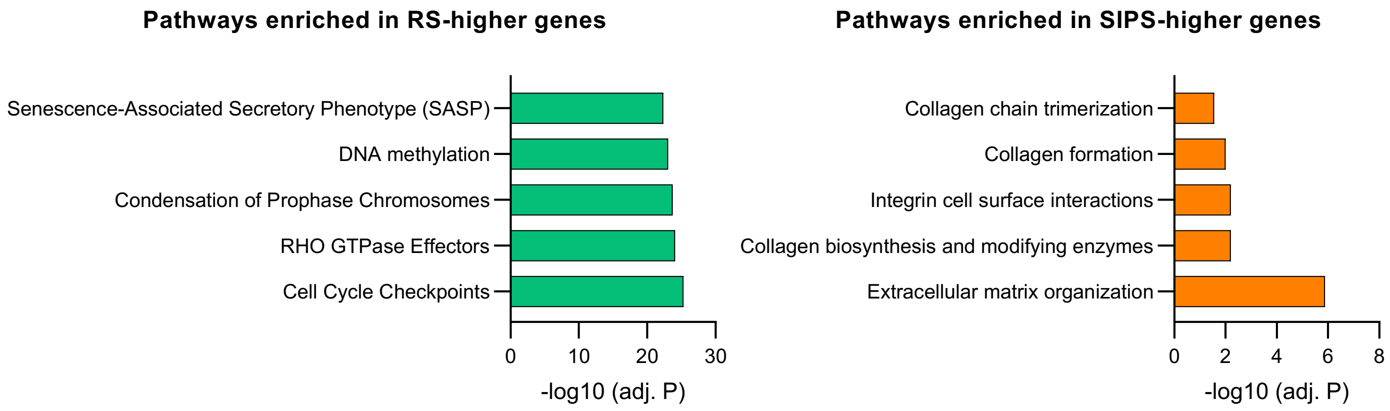

Supplement: S2 Fig — (A) Volcano plots of differentially expressed genes (DEGs) between RS- and SIPS-HUVECs were shown. (B) The top 5 enriched reactome pathways enriched in either RS- or SIPS-ECs. (PDF) [file pone.0351140.s002.pdf]
