# Supplementary material for: Replicative and stress-induced premature senescence distinctively affect the endothelial anticoagulation capacity
Source: PLoS One. 2026 Jun 9;21(6):e0351140. doi: 10.1371/journal.pone.0351140 (PMC13249167; doi:10.1371/journal.pone.0351140)
Supplement: Attachment — Submitted filename: Response to Reviewers.docx [file pone.0351140.s006.docx]

**Responses to the Reviewers’ comments**

**Reviewer #2**

**Comments**
*• ABSTRACT: Line 26 EC abrev is not explained before
• INTRODUCTION: Very well written and explicative. I suggest to add biological causes of RS and SIPS (although they are mentioned in the discussion), which is the senescence-associated secretory phenotype or how is characterized? “EC dysfunction induced by glucose, and lipids levels” is irrelevant for the rest of the manuscript, there’s not any other mention related to these metabolic changes.*

**Responses**

Thank you for the comments. We have described as “endothelial cell (EC)” in the abstract at line 26 in accordance with the comment.

Also, we have described the biological causes of RS and SIPS, and their relevance with the SASP in the Introduction of the revised manuscript, as suggested by the Reviewer.

We have deleted the description of “EC dysfunction induced by glucose, and lipids levels” as suggested by the Reviewer.

**Comments**
• METHODS: Very well explained. Linked to my previous comment: How do you address that those cells (HUVEC at passage 17-20, and SIPS -TRF2) have senescence-associated secretory phenotype? Experimental evidence should be added. Please add reference related to IVC-ligation model.

**Responses**

Thank you for the comments. We have shown that replicative senescent HUVECs highly express SASP factors in our previous reports (Nat Commun. 2020;11(1):481). Also, we have previously shown that HUVECs that overexpress the TRF2DN exhibited the enhanced expression of SASP factors; however, because SIPS induced by TRF2DN-overexpression show relatively high variation in terms of cellular senescence, we newly examined the SASP factors expression in HUVECs overexpressing TRF2DN in the current experimental settings. As shown in new Fig. S1A, SIPS-HUVECs showed enhanced SASP factors expressions comparing to control cells. We described these findings in the revised manuscript.

We have added references related to IVC ligation models in the Method section of the revised manuscript.

**Comments**
*• FIGURES: Figures are pixeled so part of the results (images) were impossible to evaluate, improving quality of all figures. Show “n” in some captions that are missing (i.e. Figure 1). Consider change the names of “Premature”, “Replication” to “SR”, “SIPS” for more logical reading.*

**Responses**

Thank you for the comments. We have uploaded Figures with improved quality. Also, we have shown “n” in all captions. As suggested by the Reviewer, we change the name of “Premature” and “Replication” to “SR” and “SIPS”, respectively.

**Comments**
*• RESULTS
o Related to Fig 1. A representative raw data from CAT can be shown next to bars? Giving more robustness and esthetics to the results, and if they are overlaid it would be better so the differences among treatments can be shown.*

**Responses**

Thank you for the comments. As suggested, we have shown a representative row data from CAT in new Fig. 1B.

**Comments**
*o Related to Fig 2. The comparisons were made aged (RS and SIPS) vs young. For this approach you should do a more appropriate analysis considering all the treatments. I strongly suggest also comparing RS vs SIPS and show through Venn’s diagram the shared upregulated and downregulated DEG so it can be clearly separated genes related to each condition. And from there do the enrichment analysis. Additionally of transcriptomics, it would be very good if you can validate it, at least some differential processes among the treatments.*

**Responses**

Thank you for the comments. In accordance with comments, we newly compared the transcriptomics between RS and SIPS. As shown in new Fig. S2, their transcriptomes showed significant difference despite shared senescent phenotypes. Pathways enriched in RS include SASP, DNA methylation, and cell cycle checkpoints, whereas extracellular-related pathways were more prominent in SIPS-ECs. These results newly described in Results of the revised manuscript.

**Comments**
*o Related to Figure 3. Could you experimentally demonstrate the mice with SIPS has induced overexpressing the dominant negative form of TRF2? As mentioned previously the figures were poor quality so I could not distinguish clearly the differences, consider also to add to the photos arrows or circles or highlight what you explain. I strongly suggest generating quantitative data from the photos (i.e. counts of cell-infiltrated so statistics can be applied to the data, and the photo will be used as a presentative picture of what is happening). Also, the neutrophils show image controls: Unspecificity of secondary antibody (basically no primary antibody), only MPO, only DAPI, and merge for each treatment.*

**Responses**

Thank you for the comments. To address Reviewer’s comments, we newly performed immunohistochemistry for the TRF2DN (that is tagged with myc-tag) using mouse lungs. As shown in new Fig.S3A, TRF2DN-myc were readily detected in vWF-positive endothelial cells in the lungs of the EC-specific SIPS mice.

In accordance with the comments, we added arrows in the photos of Fig. 3E, F, and G to highlight what we explain.

As suggested, we newly counted the cells infiltrated within the thrombi, and performed quantitative analysis. The number of infiltrated cells was significantly increased in thrombi of aged and EC-SIPS mice as compared to young and WT mice, respectively (new Fig. S3D).

Also, we performed immunohistochemistry using negative control IgG for the primary antibody as a negative control, and the results were shown in new Fig. 3G.

**Comments**
*o Related to figure 4. Are these changes observed also in the HUVEC-transcriptomics? Look for these markers in the transcriptomics (add it as supplementary if you prefer) and discuss whether these parameters change or not in both models and possible explanations, it can be really interesting.*

**Responses**

Thank you for the comments. To address the comments, we analyzed the expression of genes related to blood coagulation using the RNA-seq dataset. As shown in new Fig. S4, changes observed between lung ECs of young and EC-SIPS mice were not detected in the comparison between young and SIPS HUVECs. This variability may reflect differences in senescence extent or EC subtype (because lung ECs include ECs of aorta, vein and capillaries).

Interestingly, RS-ECs, but not SIPS-ECs, showed higher expression of tPA, THBD, and NOS3 as compared to those in young cells, which is consistent with the enhanced anticoagulation capacity in RS-ECs detected by CAT assay.

We described these new findings in the Results of the revised manuscript.

**Comments**
*• DISCUSSION: Line 386-387 is reported that a study demonstrated ‘a bit’ difference in protein expression between these cells, explain which differences. Line 396-397 and 410-411 you conclude that the data suggest SIPS is the major type of cellular senescence that occurs in aging, which data are you considering to suggest this? S3Fig, can you measure that also in the HUVEC model and add it to this supplementary? Can you explain more the limitations of this study including the model and current approaches in comparison to these? Which is the possible vivo model to extrapolate SR cells or difficulties? Biological importance of these findings or novelty of these results?*

**Responses**

Thank you for the comments. In accordance with the comments, we explained the difference in protein expression between cells with RS and SIPS, which was reported in the previous study, in the Discussion of the revised manuscript.

We revealed that both naturally aged and EC-SIPS mice showed deteriorated thrombus formation comparing to each control mice. Furthermore, histological analysis of thrombi demonstrated the enhanced neutrophil infiltration within thrombi in both naturally aged and EC-SIPS mice. Moreover, RS-ECs showed rather enhanced anticoagulation capacity compared to young cells *in vit*ro as detected by the CAT assay, which is inconsistent with the exacerbated thrombosis in naturally aged mice *in vivo*. We believe that these data collectively suggest that SIPS, rather than RS, might be the major type of cellular senescence that occurs in ECs during aging, at least with respect to their anti-coagulation capacities.

The data shown in the previous Fig. S3 was obtained using the HUVEC models. Therefore, we newly analyzed the SELE and VCAM-1 expression in lung ECs of young WT and SIPS mice. As shown in new Fig. S5B, SELE expression was significantly enhanced in lung ECs of SIPS mice, while VCAM-1 expression showed the tendency toward increase in these cells.

As suggested by the Reviewer, we described the limitation of this study including the models and current approaches in the Discussion of the revised manuscript. As long as we know, there is no in vivo model to extrapolate SR cells, at least for ECs.

**Reviewer #3**

**Comments**

*(1) a linguistic expert / an English native speaker, would be beneficial to correct recurrent grammar and syntax errors.*

**Responses**

Thank you for the comments. In accordance with the comments, we performed the English proofreading by a native speaker for this revised manuscript.

**Comments**

*(2) Most of the reagents reported lack a catalogue number*

*(3) Please provide final working concentration of the reagents used in SI unit. Dilutions are not enough to provide scientific reproducibility, since stock solutions may variate concentration*

**Responses**

Thank you for the comments. We described the catalogue number of all the reagents in the Methods of the revised manuscript. Also, we provide final working concentration of the reagents in the Methods of the revised manuscript.

**Comments**

*(4) Sequencing data, RT-qPCR results and the coagulation assay should indicate compatible results. Possible mismatch could be due to: (1) primers low specificity (2) sequencing over-amplification (3) mRNA degradation. Sequencing results could be expanded into a precise description of which genes are specific for replicative-specific senescence and stress-induced premature senescence.*

**Responses**

Thank you for the comments. To address the comments, we newly compared the transcriptomics between RS and SIPS. As shown in new Fig. S2, their transcriptomes showed significant difference despite shared senescent phenotypes. Pathways enriched in RS include SASP, DNA methylation, and cell cycle checkpoints, whereas extracellular-related pathways were more prominent in SIPS-ECs.

In our current analysis, it is difficult to determine specific genes for RS and SIPS. We strongly agree that identifying genes and/or gene set specific for RS and SIPS is very important and valuable to promote the senescence research. We described these new findings and important comments in the Results of the revised manuscript.
