# Supplementary material for: Replicative and stress-induced premature senescence distinctively affect the endothelial anticoagulation capacity
Source: PLoS One. 2026 Jun 9;21(6):e0351140. doi: 10.1371/journal.pone.0351140 (PMC13249167; doi:10.1371/journal.pone.0351140)
Supplement: Attachment — Submitted filename: Response_to_Reviewers_auresp_2.docx [file pone.0351140.s007.docx]

**Response to Reviewers’ comments**

**Reviewer #2:**

**Comments**

*I appreciate the authors' efforts in addressing the previous comments.
The authors have addressed all technical and methodological concerns.*

**Response**

We appreciate the Reviewer’s positive evaluation.

**Reviewer #3**

**Comments**

*Regarding comment (1). The language of the paper was substantially improved. However, a few minor errors remain. E.g.:
line 49: “According to the World Health Organization, approximately 18 million mortality in 2019 were attributed to cardiovascular diseases, accounts for 32% of all global mortality”.
The term “mortality” should be substituted with “deaths” and “accounts for” with “accounting for”.
line 146: “The mice were fed chow with ad libitum access to water and food and maintained under a 12-h light/12-h dark cycle”. The wording is redundant, please consider rephrasing it as: “Mice were given ad libitum access to water and food under a 12-h light/12-h dark cycle”.
Issue partially resolved; a few minor corrections still need to be made.*

**Response**

We appreciate the Reviewer’s positive evaluation.

We additionally made a few corrections in the revised manuscript as suggested by the Reviewer.

**Comments**
*Regarding comment (2). Antibody and reagent catalogue numbers were successfully updated, allowing scientific reproducibility. In only one case was it impossible to find a match: FluCa Kit (#86322). In the Stago online catalogue (English and German versions), I found the corresponding article number #86197 for the FluCa Kit and from Fisher Scientific (Diagnostica Stago as supplier) the following catalogue number for the same kit: NC9074822. Please verify whether there is a discrepancy between the printed catalogue you possess and the publicly available one, or if it was simply a “copy-paste” error.
Issue partially resolved; please verify FluCa Kit catalogue number.*

**Response**

We have checked the catalogue number of FluCa Kit that we used, and confirmed that the catalogue number labeled on the box is #86322 as shown below. According to the distributor, they changed the catalogue number from #86322 to #86197 on November, 2025 when they changed reagent containers from glass to plastic vial. Because we are not sure if the current FluCa kit (#86197) is identical to the one we used (#86322), we described the catalogue number #86322 as it was for the kit we used in the experiments.

**Comments**
*Regarding comment (3). The final concentrations of reagents were reported in SI units, ensuring the reproducibility of the experiments. Issue resolved.
Regarding comment (4). I appreciate the supplementary S2 Fig.: statistical significance was reached. I would suggest (it is optional, not mandatory) to plot on the volcano plot (S2A Fig.) the labels of the genes you already mention in supplementary materials (S1A, S3A, S4 and S5 Fig.). It is not a problem if some genes exhibit a P adj > 0.05, it is expected with n =3. Issue resolved*

**Response**

We appreciate the Reviewer’s positive evaluation.

In accordance with the comments, we newly plotted the labels of genes mentioned in this manuscript on the volcano plot (new S2A Fig.).
