# Supplementary material for: Replicative and stress-induced premature senescence distinctively affect the endothelial anticoagulation capacity
Source: PLoS One. 2026 Jun 9;21(6):e0351140. doi: 10.1371/journal.pone.0351140 (PMC13249167; doi:10.1371/journal.pone.0351140)
Supplement: Peer review history

# Peer review: Decision Letter 0

**DOI:** 10.1371/journal.pone.0351140.r001

**Authors:** Tomasz Kaminski

16 Feb 2026

PONE-D-26-02941

Replicative and stress-induced premature senescence distinctively affect the endothelial anticoagulation capacity

PLOS One

Dear Dr. Ikeda,

Thank you for submitting your manuscript to PLOS ONE. After careful consideration, we feel that it has merit but does not fully meet PLOS ONE’s publication criteria as it currently stands. Therefore, we invite you to submit a revised version of the manuscript that addresses the points raised during the review process.

Please submit your revised manuscript by Apr 02 2026 11:59PM. If you will need more time than this to complete your revisions, please reply to this message or contact the journal office at plosone@plos.org. When you're ready to submit your revision, log on to https://www.editorialmanager.com/pone/ and select the 'Submissions Needing Revision' folder to locate your manuscript file.

Please include the following items when submitting your revised manuscript:

- A letter that responds to each point raised by the academic editor and reviewer(s). You should upload this letter as a separate file labeled 'Response to Reviewers'.
- A marked-up copy of your manuscript that highlights changes made to the original version. You should upload this as a separate file labeled 'Revised Manuscript with Track Changes'.
- An unmarked version of your revised paper without tracked changes. You should upload this as a separate file labeled 'Manuscript'.

If you would like to make changes to your financial disclosure, please include your updated statement in your cover letter. Guidelines for resubmitting your figure files are available below the reviewer comments at the end of this letter.

If applicable, we recommend that you deposit your laboratory protocols in protocols.io to enhance the reproducibility of your results. Protocols.io assigns your protocol its own identifier (DOI) so that it can be cited independently in the future. For instructions see: https://journals.plos.org/plosone/s/submission-guidelines#loc-laboratory-protocols. Additionally, PLOS ONE offers an option for publishing peer-reviewed Lab Protocol articles, which describe protocols hosted on protocols.io. Read more information on sharing protocols at https://plos.org/protocols?utm_medium=editorial-email&utm_source=authorletters&utm_campaign=protocols.

We look forward to receiving your revised manuscript.

Kind regards,

Tomasz W. Kaminski

Academic Editor

PLOS One

**Journal Requirements:**

When submitting your revision, we need you to address these additional requirements.

1. Please ensure that your manuscript meets PLOS ONE's style requirements, including those for file naming. The PLOS ONE style templates can be found at

https://journals.plos.org/plosone/s/file?id=wjVg/PLOSOne_formatting_sample_main_body.pdf and

https://journals.plos.org/plosone/s/file?id=ba62/PLOSOne_formatting_sample_title_authors_affiliations.pdf

2. We note that the grant information you provided in the ‘Funding Information’ and ‘Financial Disclosure’ sections do not match.

When you resubmit, please ensure that you provide the correct grant numbers for the awards you received for your study in the ‘Funding Information’ section.

3. Thank you for stating the following financial disclosure:

“KI received the Grant from JSPS KAKENHI (Grant Numbers JP22H03517).”

Please state what role the funders took in the study.  If the funders had no role, please state: "The funders had no role in study design, data collection and analysis, decision to publish, or preparation of the manuscript."

If this statement is not correct you must amend it as needed.

Please include this amended Role of Funder statement in your cover letter; we will change the online submission form on your behalf.

4. Please expand the acronym “JSPS” (as indicated in your financial disclosure) so that it states the name of your funders in full.

This information should be included in your cover letter; we will change the online submission form on your behalf.

5. Please note that your Data Availability Statement is currently missing the repository name and a direct link to access each database. If your manuscript is accepted for publication, you will be asked to provide these details on a very short timeline. We therefore suggest that you provide this information now, though we will not hold up the peer review process if you are unable.

6. Please upload a new copy of Figures 1, 2, 3, and 4 as the detail is not clear. Please follow the link for more information:  https://journals.plos.org/plosone/s/figures

7. If the reviewer comments include a recommendation to cite specific previously published works, please review and evaluate these publications to determine whether they are relevant and should be cited. There is no requirement to cite these works unless the editor has indicated otherwise.

**Additional Editor Comments:**

Dear Authors,

Thank you for submitting your manuscript.

While the study addresses an important and timely question and presents promising and potentially impactful findings, several substantial issues related to mechanistic validation, statistical rigor, and clarity of data presentation need to be addressed before the work can be considered for publication. In particular, the reviewers raised concerns regarding the biological characterization of the senescence models, transcriptomic comparisons between RS and SIPS, reproducibility, and the need for improved figure quality and methodological transparency; therefore, we invite you to revise your manuscript accordingly.

Best regards,

Tomasz W Kaminski

[Note: HTML markup is below. Please do not edit.]

Reviewers' comments:

Reviewer's Responses to Questions

**Comments to the Author**

1. Is the manuscript technically sound, and do the data support the conclusions?

The manuscript must describe a technically sound piece of scientific research with data that supports the conclusions. Experiments must have been conducted rigorously, with appropriate controls, replication, and sample sizes. The conclusions must be drawn appropriately based on the data presented.

Reviewer #1: Yes

Reviewer #2: Yes

Reviewer #3: Yes

**********

2. Has the statistical analysis been performed appropriately and rigorously?

Reviewer #1: Yes

Reviewer #2: Yes

Reviewer #3: Yes

**********

3. Have the authors made all data underlying the findings in their manuscript fully available?

The PLOS Data policy requires authors to make all data underlying the findings described in their manuscript fully available without restriction, with rare exception (please refer to the Data Availability Statement in the manuscript PDF file). The data should be provided as part of the manuscript or its supporting information, or deposited to a public repository. For example, in addition to summary statistics, the data points behind means, medians and variance measures should be available. If there are restrictions on publicly sharing data—e.g. participant privacy or use of data from a third party—those must be specified.

Reviewer #1: Yes

Reviewer #2: Yes

Reviewer #3: Yes

**********

4. Is the manuscript presented in an intelligible fashion and written in standard English?

PLOS ONE does not copyedit accepted manuscripts, so the language in submitted articles must be clear, correct, and unambiguous. Any typographical or grammatical errors should be corrected at revision, so please note any specific errors here.

Reviewer #1: Yes

Reviewer #2: Yes

Reviewer #3: No

**********

5. Review Comments to the Author

Please use the space provided to explain your answers to the questions above. You may also include additional comments for the author, including concerns about dual publication, research ethics, or publication ethics. (Please upload your review as an attachment if it exceeds 20,000 characters)

Reviewer #1: This study convincingly demonstrates that **replicative senescence (RS)** and **stress-induced premature senescence (SIPS)** exert fundamentally different effects on endothelial anticoagulation, resolving an important ambiguity in vascular aging research. While RS paradoxically enhances anticoagulant capacity in vitro by reducing thrombin generation, this protective signal fails to translate in vivo, where aging is instead associated with exacerbated thrombosis—an effect recapitulated by EC-specific SIPS models. Through integrated CAT assays, RNA-seq, and murine IVC ligation experiments, the authors show that SIPS, rather than RS, mirrors the transcriptional, histological, and functional coagulation phenotypes observed in natural aging, particularly via altered platelet adhesion, immune infiltration, and neutrophil-driven thrombus architecture. A key strength lies in the mechanistic triangulation between in vitro endothelial behavior, in vivo thrombosis, and gene expression concordance; however, the reliance on lung-derived ECs and acute thrombosis models may limit extrapolation to other vascular beds and chronic disease states. Overall, the work provides strong evidence that **SIPS is the dominant, pathophysiologically relevant senescence program in age-related endothelial thrombogenicity**, with important implications for senescence-targeted vascular therapies.

Comments

1. Transparency and Reproducibility

The authors should provide full open-source code, pretrained models, and data preprocessing scripts in a publicly accessible repository. Including reproducible Jupyter notebooks for training, evaluation, and ablation would demonstrate commitment to transparency.

2. Comprehensive Dataset and Method Details

Expand dataset descriptions with detailed information about patient/sample demographics, imaging modalities, annotation standards, and multi-center data harmonization. Include explicit preprocessing and augmentation details to improve reproducibility.

3. Robust Experimental Design and Statistical Rigor

Implement stratified k-fold cross-validation or repeated random splits with detailed fold-wise metrics. Present statistical significance testing (e.g., paired t-test, Wilcoxon) for key results and detailed per-class performance breakdowns.

4. Expanded Explainability Approaches

Integrate additional explainability frameworks (e.g., Grad-CAM, LIME, SHAP) with relevant qualitative and quantitative analyses. Include case studies explaining both successes and errors to support clinical interpretability.

5. Ablation and Component Impact Analysis

Provide detailed ablations for model components, data preprocessing steps, and optimization algorithms. Report both quantitative metrics and qualitative results indicating the contribution of each component.

6. Broader Benchmarking

Benchmark against state-of-the-art deep learning methods relevant to the problem, including attention-based, transformer, and hybrid architectures. Include efficiency comparisons relevant for clinical use.

7. Handling Imbalanced Data and Rare Cases

Discuss strategies employed for imbalanced data or rare conditions, reporting per-class metrics, and experiments with weighted losses or data augmentation.

8. Clinical Integration and Translation

Elaborate on considerations for real-world deployment in clinical settings, regulatory challenges, device heterogeneity, and ethical implications. Propose plans for external, multicenter, or prospective validation.

9. Enhanced Literature Integration

Expand literature review to include recent advances and foundational works in biomedical AI, especially explicability, federated learning, and clinical translation, citing:

https://www.sciencedirect.com/science/article/pii/S1476927125000283

https://link.springer.com/article/10.1007/s11831-025-10255-2

https://www.sciencedirect.com/science/article/pii/S1476927125001604?via%3Dihub

https://www.nature.com/articles/s41598-025-12602-6

https://pubmed.ncbi.nlm.nih.gov/40751377/

https://www.nature.com/articles/s41598-025-11574-x

https://link.springer.com/article/10.1007/s11831-025-10315-7

https://www.nature.com/articles/s41598-025-14333-0

https://link.springer.com/article/10.1007/s11831-025-10376-8?utm_source=rct_congratemailt&utm_medium=email&utm_campaign=nonoa_20251002&utm_content=10.1007%2Fs11831-025-10376-8

https://link.springer.com/article/10.1007/s11831-025-10379-5

https://link.springer.com/article/10.1007/s11831-025-10414-5

https://link.springer.com/article/10.1007/s11831-025-10411-8

https://www.sciencedirect.com/science/article/pii/S1746809425013631?r

https://journals.tubitak.gov.tr/biology/vol49/iss5/9/

10. Result Presentation and Visualization

Enhance result presentation with detailed confusion matrices, ROC and PR curves, attention heatmaps, and error analysis for challenging cases.

11. Methodological Clarity

Clearly detail all model hyperparameters, training schedules, optimizer choices, loss functions, and software/hardware environments to facilitate exact replication.

12. Future Research Directions

Discuss opportunities for multi-modal integration, real-time deployment, federated learning, longitudinal studies, and prospective clinical trials to accelerate translation.

Reviewer #2: This study is very well written, easy to follow and explores an interesting and potentially important question regarding how distinct forms of endothelial senescence differentially impact coagulation and thrombosis. The experimental framework is broad; the aim is clear and is approached in vitro and in vivo with different models. However, some issues need to be addressed to strengthen the manuscript. Below my comments by section

• ABSTRACT: Line 26 EC abrev is not explained before

• INTRODUCTION: Very well written and explicative. I suggest to add biological causes of RS and SIPS (although they are mentioned in the discussion), which is the senescence-associated secretory phenotype or how is characterized? “EC dysfunction induced by glucose, and lipids levels” is irrelevant for the rest of the manuscript, there’s not any other mention related to these metabolic changes.

• METHODS: Very well explained. Linked to my previous comment: How do you address that those cells (HUVEC at passage 17-20, and SIPS -TRF2) have senescence-associated secretory phenotype? Experimental evidence should be added. Please add reference related to IVC-ligation model.

• FIGURES: Figures are pixeled so part of the results (images) were impossible to evaluate, improving quality of all figures. Show “n” in some captions that are missing (i.e. Figure 1). Consider change the names of “Premature”, “Replication” to “SR”, “SIPS” for more logical reading.

• RESULTS

o Related to Fig 1. A representative raw data from CAT can be shown next to bars? Giving more robustness and esthetics to the results, and if they are overlaid it would be better so the differences among treatments can be shown.

o Related to Fig 2. The comparisons were made aged (RS and SIPS) vs young. For this approach you should do a more appropriate analysis considering all the treatments. I strongly suggest also comparing RS vs SIPS and show through Venn’s diagram the shared upregulated and downregulated DEG so it can be clearly separated genes related to each condition. And from there do the enrichment analysis. Additionally of transcriptomics, it would be very good if you can validate it, at least some differential processes among the treatments.

o Related to Figure 3. Could you experimentally demonstrate the mice with SIPS has induced overexpressing the dominant negative form of TRF2? As mentioned previously the figures were poor quality so I could not distinguish clearly the differences, consider also to add to the photos arrows or circles or highlight what you explain. I strongly suggest generating quantitative data from the photos (i.e. counts of cell-infiltrated so statistics can be applied to the data, and the photo will be used as a presentative picture of what is happening). Also, the neutrophils show image controls: Unspecificity of secondary antibody (basically no primary antibody), only MPO, only DAPI, and merge for each treatment.

o Related to figure 4. Are these changes observed also in the HUVEC-transcriptomics? Look for these markers in the transcriptomics (add it as supplementary if you prefer) and discuss whether these parameters change or not in both models and possible explanations, it can be reallt interesting.

• DISCUSSION: Line 386-387 is reported that a study demonstrated ‘a bit’ difference in protein expression between these cells, explain which differences. Line 396-397 and 410-411 you conclude that the data suggest SIPS is the major type of cellular senescence that occurs in aging, which data are you considering to suggest this? S3Fig, can you measure that also in the HUVEC model and add it to this supplementary? Can you explain more the limitations of this study including the model and current approaches in comparison to these? Which is the possible vivo model to extrapolate SR cells or difficulties? Biological importance of these findings or novelty of these results?

Reviewer #3: The work presents a good logical work-flow, with solid data and clear results. The authors identified that aging actively influences the anticoagulation capacity of endothelial cells and more specifically that, in cultivated cell cultures, aged endothelial cells under replicative stress showed a stronger effect than endothelial cells which underwent stress-induced premature senescence. This result was validated through RT-qPCR. The principal component analysis reported in Fig. 2 shows samples distributed along the 3 different conditions: it is usually a good sign, indicating a successful sequencing.

Although the results are promising, some minor corrections should be taken into consideration: (1) a linguistic expert / an English native speaker, would be beneficial to correct recurrent grammar and syntax errors. (2) Most of the reagents reported lack a catalogue number (3) Please provide final working concentration of the reagents used in SI unit. Dilutions are not enough to provide scientific reproducibility, since stock solutions may variate concentration (4) Sequencing data, RT-qPCR results and the coagulation assay should indicate compatible results. Possible mismatch could be due to: (1) primers low specificity (2) sequencing over-amplification (3) mRNA degradation. Sequencing results could be expanded into a precise description of which genes are specific for replicative-specific senescence and stress-induced premature senescence.

I think that the paper should be accepted, once the authors performed the aforementioned corrections. The language seems to be the main problem of this work. Considering that both RT-qPCR and coagulation-assay point in the same direction, I would consider the results as valid.

**********

6. PLOS authors have the option to publish the peer review history of their article (what does this mean?). If published, this will include your full peer review and any attached files.

If you choose “no”, your identity will remain anonymous but your review may still be made public.

**Do you want your identity to be public for this peer review?** For information about this choice, including consent withdrawal, please see our Privacy Policy.

Reviewer #1: No

Reviewer #2: **Yes:** Jose R Pittaluga-Villarreal

Reviewer #3: **Yes:** Adriano Sanna

**********

[NOTE: If reviewer comments were submitted as an attachment file, they will be attached to this email and accessible via the submission site. Please log into your account, locate the manuscript record, and check for the action link "View Attachments". If this link does not appear, there are no attachment files.]

To ensure your figures meet our technical requirements, please review our figure guidelines: https://journals.plos.org/plosone/s/figures

You may also use PLOS’s free figure tool, NAAS, to help you prepare publication quality figures: https://journals.plos.org/plosone/s/figures#loc-tools-for-figure-preparation.

NAAS will assess whether your figures meet our technical requirements by comparing each figure against our figure specifications.

# Author comment: Author response to Decision Letter 1

**DOI:** 10.1371/journal.pone.0351140.r002

7 May 2026

Responses to the Reviewers’ comments

Reviewer #2

Comments

• ABSTRACT: Line 26 EC abrev is not explained before

• INTRODUCTION: Very well written and explicative. I suggest to add biological causes of RS and SIPS (although they are mentioned in the discussion), which is the senescence-associated secretory phenotype or how is characterized? “EC dysfunction induced by glucose, and lipids levels” is irrelevant for the rest of the manuscript, there’s not any other mention related to these metabolic changes.

Responses

Thank you for the comments. We have described as “endothelial cell (EC)” in the abstract at line 26 in accordance with the comment.

Also, we have described the biological causes of RS and SIPS, and their relevance with the SASP in the Introduction of the revised manuscript, as suggested by the Reviewer.

We have deleted the description of “EC dysfunction induced by glucose, and lipids levels” as suggested by the Reviewer.

Comments

• METHODS: Very well explained. Linked to my previous comment: How do you address that those cells (HUVEC at passage 17-20, and SIPS -TRF2) have senescence-associated secretory phenotype? Experimental evidence should be added. Please add reference related to IVC-ligation model.

Responses

Thank you for the comments. We have shown that replicative senescent HUVECs highly express SASP factors in our previous reports (Nat Commun. 2020;11(1):481). Also, we have previously shown that HUVECs that overexpress the TRF2DN exhibited the enhanced expression of SASP factors; however, because SIPS induced by TRF2DN-overexpression show relatively high variation in terms of cellular senescence, we newly examined the SASP factors expression in HUVECs overexpressing TRF2DN in the current experimental settings. As shown in new Fig. S1A, SIPS-HUVECs showed enhanced SASP factors expressions comparing to control cells. We described these findings in the revised manuscript.

We have added references related to IVC ligation models in the Method section of the revised manuscript.

Comments

• FIGURES: Figures are pixeled so part of the results (images) were impossible to evaluate, improving quality of all figures. Show “n” in some captions that are missing (i.e. Figure 1). Consider change the names of “Premature”, “Replication” to “SR”, “SIPS” for more logical reading.

Responses

Thank you for the comments. We have uploaded Figures with improved quality. Also, we have shown “n” in all captions. As suggested by the Reviewer, we change the name of “Premature” and “Replication” to “SR” and “SIPS”, respectively.

Comments

• RESULTS

o Related to Fig 1. A representative raw data from CAT can be shown next to bars? Giving more robustness and esthetics to the results, and if they are overlaid it would be better so the differences among treatments can be shown.

Responses

Thank you for the comments. As suggested, we have shown a representative row data from CAT in new Fig. 1B.

Comments

o Related to Fig 2. The comparisons were made aged (RS and SIPS) vs young. For this approach you should do a more appropriate analysis considering all the treatments. I strongly suggest also comparing RS vs SIPS and show through Venn’s diagram the shared upregulated and downregulated DEG so it can be clearly separated genes related to each condition. And from there do the enrichment analysis. Additionally of transcriptomics, it would be very good if you can validate it, at least some differential processes among the treatments.

Responses

Thank you for the comments. In accordance with comments, we newly compared the transcriptomics between RS and SIPS. As shown in new Fig. S2, their transcriptomes showed significant difference despite shared senescent phenotypes. Pathways enriched in RS include SASP, DNA methylation, and cell cycle checkpoints, whereas extracellular-related pathways were more prominent in SIPS-ECs. These results newly described in Results of the revised manuscript.

Comments

o Related to Figure 3. Could you experimentally demonstrate the mice with SIPS has induced overexpressing the dominant negative form of TRF2? As mentioned previously the figures were poor quality so I could not distinguish clearly the differences, consider also to add to the photos arrows or circles or highlight what you explain. I strongly suggest generating quantitative data from the photos (i.e. counts of cell-infiltrated so statistics can be applied to the data, and the photo will be used as a presentative picture of what is happening). Also, the neutrophils show image controls: Unspecificity of secondary antibody (basically no primary antibody), only MPO, only DAPI, and merge for each treatment.

Responses

Thank you for the comments. To address Reviewer’s comments, we newly performed immunohistochemistry for the TRF2DN (that is tagged with myc-tag) using mouse lungs. As shown in new Fig.S3A, TRF2DN-myc were readily detected in vWF-positive endothelial cells in the lungs of the EC-specific SIPS mice.

In accordance with the comments, we added arrows in the photos of Fig. 3E, F, and G to highlight what we explain.

As suggested, we newly counted the cells infiltrated within the thrombi, and performed quantitative analysis. The number of infiltrated cells was significantly increased in thrombi of aged and EC-SIPS mice as compared to young and WT mice, respectively (new Fig. S3D).

Also, we performed immunohistochemistry using negative control IgG for the primary antibody as a negative control, and the results were shown in new Fig. 3G.

Comments

o Related to figure 4. Are these changes observed also in the HUVEC-transcriptomics? Look for these markers in the transcriptomics (add it as supplementary if you prefer) and discuss whether these parameters change or not in both models and possible explanations, it can be really interesting.

Responses

Thank you for the comments. To address the comments, we analyzed the expression of genes related to blood coagulation using the RNA-seq dataset. As shown in new Fig. S4, changes observed between lung ECs of young and EC-SIPS mice were not detected in the comparison between young and SIPS HUVECs. This variability may reflect differences in senescence extent or EC subtype (because lung ECs include ECs of aorta, vein and capillaries).

Interestingly, RS-ECs, but not SIPS-ECs, showed higher expression of tPA, THBD, and NOS3 as compared to those in young cells, which is consistent with the enhanced anticoagulation capacity in RS-ECs detected by CAT assay.

We described these new findings in the Results of the revised manuscript.

Comments

• DISCUSSION: Line 386-387 is reported that a study demonstrated ‘a bit’ difference in protein expression between these cells, explain which differences. Line 396-397 and 410-411 you conclude that the data suggest SIPS is the major type of cellular senescence that occurs in aging, which data are you considering to suggest this? S3Fig, can you measure that also in the HUVEC model and add it to this supplementary? Can you explain more the limitations of this study including the model and current approaches in comparison to these? Which is the possible vivo model to extrapolate SR cells or difficulties? Biological importance of these findings or novelty of these results?

Responses

Thank you for the comments. In accordance with the comments, we explained the difference in protein expression between cells with RS and SIPS, which was reported in the previous study, in the Discussion of the revised manuscript.

We revealed that both naturally aged and EC-SIPS mice showed deteriorated thrombus formation comparing to each control mice. Furthermore, histological analysis of thrombi demonstrated the enhanced neutrophil infiltration within thrombi in both naturally aged and EC-SIPS mice. Moreover, RS-ECs showed rather enhanced anticoagulation capacity compared to young cells in vitro as detected by the CAT assay, which is inconsistent with the exacerbated thrombosis in naturally aged mice in vivo. We believe that these data collectively suggest that SIPS, rather than RS, might be the major type of cellular senescence that occurs in ECs during aging, at least with respect to their anti-coagulation capacities.

The data shown in the previous Fig. S3 was obtained using the HUVEC models. Therefore, we newly analyzed the SELE and VCAM-1 expression in lung ECs of young WT and SIPS mice. As shown in new Fig. S5B, SELE expression was significantly enhanced in lung ECs of SIPS mice, while VCAM-1 expression showed the tendency toward increase in these cells.

As suggested by the Reviewer, we described the limitation of this study including the models and current approaches in the Discussion of the revised manuscript. As long as we know, there is no in vivo model to extrapolate SR cells, at least for ECs.

Reviewer #3

Comments

(1) a linguistic expert / an English native speaker, would be beneficial to correct recurrent grammar and syntax errors.

Responses

Thank you for the comments. In accordance with the comments, we performed the English proofreading by a native speaker for this revised manuscript.

Comments

(2) Most of the reagents reported lack a catalogue number

(3) Please provide final working concentration of the reagents used in SI unit. Dilutions are not enough to provide scientific reproducibility, since stock solutions may variate concentration

Responses

Thank you for the comments. We described the catalogue number of all the reagents in the Methods of the revised manuscript. Also, we provide final working concentration of the reagents in the Methods of the revised manuscript.

Comments

(4) Sequencing data, RT-qPCR results and the coagulation assay should indicate compatible results. Possible mismatch could be due to: (1) primers low specificity (2) sequencing over-amplification (3) mRNA degradation. Sequencing results could be expanded into a precise description of which genes are specific for replicative-specific senescence and stress-induced premature senescence.

Responses

Thank you for the comments. To address the comments, we newly compared the transcriptomics between RS and SIPS. As shown in new Fig. S2, their transcriptomes showed significant difference despite shared senescent phenotypes. Pathways enriched in RS include SASP, DNA methylation, and cell cycle checkpoints, whereas extracellular-related pathways were more prominent in SIPS-ECs.

In our current analysis, it is difficult to determine specific genes for RS and SIPS. We strongly agree that identifying genes and/or gene set specific for RS and SIPS is very important and valuable to promote the senescence research. We described these new findings and important comments in the Results of the revised manuscript.

Attachment Submitted filename: Response to Reviewers.docx

# Peer review: Decision Letter 1

**DOI:** 10.1371/journal.pone.0351140.r003

**Authors:** Tomasz Kaminski

18 May 2026

PONE-D-26-02941R1

Replicative and stress-induced premature senescence distinctively affect the endothelial anticoagulation capacity

PLOS One

Dear Dr. Ikeda,

Thank you for submitting your manuscript to PLOS ONE. After careful consideration, we feel that it has merit but does not fully meet PLOS ONE’s publication criteria as it currently stands. Therefore, we invite you to submit a revised version of the manuscript that addresses the points raised during the review process.

Please submit your revised manuscript by Jul 02 2026 11:59PM. If you will need more time than this to complete your revisions, please reply to this message or contact the journal office at plosone@plos.org. When you're ready to submit your revision, log on to https://www.editorialmanager.com/pone/ and select the 'Submissions Needing Revision' folder to locate your manuscript file.

Please include the following items when submitting your revised manuscript:

- A letter that responds to each point raised by the academic editor and reviewer(s). You should upload this letter as a separate file labeled 'Response to Reviewers'.
- A marked-up copy of your manuscript that highlights changes made to the original version. You should upload this as a separate file labeled 'Revised Manuscript with Track Changes'.
- An unmarked version of your revised paper without tracked changes. You should upload this as a separate file labeled 'Manuscript'.

If you would like to make changes to your financial disclosure, please include your updated statement in your cover letter. Guidelines for resubmitting your figure files are available below the reviewer comments at the end of this letter.

If applicable, we recommend that you deposit your laboratory protocols in protocols.io to enhance the reproducibility of your results. Protocols.io assigns your protocol its own identifier (DOI) so that it can be cited independently in the future. For instructions see: https://journals.plos.org/plosone/s/submission-guidelines#loc-laboratory-protocols. Additionally, PLOS ONE offers an option for publishing peer-reviewed Lab Protocol articles, which describe protocols hosted on protocols.io. Read more information on sharing protocols at https://plos.org/protocols?utm_medium=editorial-email&utm_source=authorletters&utm_campaign=protocols.

As the corresponding author, your ORCID iD is verified in the submission system and will appear in the published article. PLOS supports the use of ORCID, and we encourage all coauthors to register for an ORCID iD and use it as well. Please encourage your coauthors to verify their ORCID iD within the submission system before final acceptance, as unverified ORCID iDs will not appear in the published article. *Only* the individual author can complete the verification step; PLOS staff *cannot* verify ORCID iDs on behalf of authors.

We look forward to receiving your revised manuscript.

Kind regards,

Tomasz W. Kaminski

Academic Editor

PLOS One

Journal Requirements:

1. If the reviewer comments include a recommendation to cite specific previously published works, please review and evaluate these publications to determine whether they are relevant and should be cited. There is no requirement to cite these works unless the editor has indicated otherwise.

2. Please review your reference list to ensure that it is complete and correct. If you have cited papers that have been retracted, please include the rationale for doing so in the manuscript text, or remove these references and replace them with relevant current references. Any changes to the reference list should be mentioned in the rebuttal letter that accompanies your revised manuscript. If you need to cite a retracted article, indicate the article’s retracted status in the References list and also include a citation and full reference for the retraction notice.

**Additional Editor Comments:**

Dear Authors,

Thank you for the revised version of the manuscript. The study has significantly improved, and the major concerns raised previously were appropriately addressed.

Only a few minor points remain:

a) Please implement the remaining minor language corrections:

Line 49:

replace “mortality” with “deaths”

replace “accounts for” with “accounting for”

Line 146:

please consider simplifying the sentence to:

“Mice were given ad libitum access to water and food under a 12-h light/12-h dark cycle.”

b) Please verify the catalogue number for the FluCa Kit. I was unable to identify the reported number (#86322) in publicly accessible catalogues. I found alternative identifiers:

Stago: #86197

Fisher Scientific: NC9074822

Please confirm whether this discrepancy reflects a different/internal catalogue version or simply a typographical error.

The optional suggestion regarding additional gene labels on the volcano plot may improve readability, but this is not mandatory.

Finally, several comments raised in the additional review by Reviewer 1 appear generic and not directly applicable to the scope of the present experimental study (e.g., requests related to deep learning frameworks, pretrained models, Grad-CAM, SHAP, federated learning, and AI benchmarking). Therefore, there is no need to incorporate or cite the suggested AI-focused references since they are not relevant to the scientific context of the manuscript. Please ignore this review.

Best regards,

Tomasz W Kaminski

[Note: HTML markup is below. Please do not edit.]

Reviewers' comments:

Reviewer's Responses to Questions

**Comments to the Author**

1. If the authors have adequately addressed your comments raised in a previous round of review and you feel that this manuscript is now acceptable for publication, you may indicate that here to bypass the “Comments to the Author” section, enter your conflict of interest statement in the “Confidential to Editor” section, and submit your "Accept" recommendation.

Reviewer #1: All comments have been addressed

Reviewer #2: All comments have been addressed

Reviewer #3: All comments have been addressed

**********

2. Is the manuscript technically sound, and do the data support the conclusions?

The manuscript must describe a technically sound piece of scientific research with data that supports the conclusions. Experiments must have been conducted rigorously, with appropriate controls, replication, and sample sizes. The conclusions must be drawn appropriately based on the data presented.

Reviewer #1: No

Reviewer #2: Yes

Reviewer #3: Yes

**********

3. Has the statistical analysis been performed appropriately and rigorously?

Reviewer #1: No

Reviewer #2: Yes

Reviewer #3: Yes

**********

4. Have the authors made all data underlying the findings in their manuscript fully available?

The PLOS Data policy requires authors to make all data underlying the findings described in their manuscript fully available without restriction, with rare exception (please refer to the Data Availability Statement in the manuscript PDF file). The data should be provided as part of the manuscript or its supporting information, or deposited to a public repository. For example, in addition to summary statistics, the data points behind means, medians and variance measures should be available. If there are restrictions on publicly sharing data—e.g. participant privacy or use of data from a third party—those must be specified.

Reviewer #1: Yes

Reviewer #2: Yes

Reviewer #3: Yes

**********

5. Is the manuscript presented in an intelligible fashion and written in standard English?

PLOS ONE does not copyedit accepted manuscripts, so the language in submitted articles must be clear, correct, and unambiguous. Any typographical or grammatical errors should be corrected at revision, so please note any specific errors here.

Reviewer #1: Yes

Reviewer #2: Yes

Reviewer #3: No

**********

6. Review Comments to the Author

Please use the space provided to explain your answers to the questions above. You may also include additional comments for the author, including concerns about dual publication, research ethics, or publication ethics. (Please upload your review as an attachment if it exceeds 20,000 characters)

Reviewer #1: The manuscript attempts to delineate the differential roles of replicative senescence (RS) and stress-induced premature senescence (SIPS) in endothelial anticoagulation and thrombosis; however, its central conclusions are not convincingly supported by the presented data. The claim that SIPS represents the predominant senescence phenotype driving age-related thrombosis is largely inferential rather than causally demonstrated, as no direct quantification or lineage-specific validation of RS versus SIPS populations in vivo is provided. Instead, the authors extrapolate from disconnected observations—namely, that RS enhances anticoagulation in vitro while aged and SIPS mouse models exhibit increased thrombosis—which constitutes a weak correlative framework rather than mechanistic evidence. This issue is compounded by a fundamental inconsistency between in vitro and in vivo findings that remains unresolved, raising concerns about the biological relevance and translational validity of the experimental models. Moreover, the TRF2DN-based induction of SIPS is itself problematic, as it relies on telomere dysfunction, thereby blurring the conceptual distinction between RS and SIPS and limiting its physiological relevance as a model of stress-induced senescence.

Methodologically, the study lacks sufficient depth to support its claims. Although RNA sequencing identifies broad transcriptional differences, the analysis remains superficial, failing to identify key regulatory drivers, validate critical pathways, or establish causal links between gene expression changes and functional phenotypes. The absence of functional validation experiments—such as gene perturbation, platelet interaction assays, or fibrinolytic activity measurements—further weakens the mechanistic interpretation. Additionally, statistical rigor is limited, with small sample sizes and the use of less stringent post hoc testing, potentially inflating significance. The biological interpretation of pathway enrichment results appears speculative, particularly in linking distinct transcriptional signatures to coagulation mechanisms without experimental confirmation. Similarly, observations of increased neutrophil infiltration in thrombi are purely descriptive and not functionally interrogated, leaving a major aspect of the proposed mechanism unexplored.

Finally, the manuscript suffers from conceptual and presentation issues that further undermine its impact. The distinction between RS and SIPS remains operational rather than biologically defined, and the discussion selectively emphasizes findings that support the authors’ hypothesis while overlooking alternative explanations such as mixed senescence states or systemic aging effects. Language and clarity issues persist despite revision, and the translational relevance of the findings is limited due to the lack of human validation or clinical correlation. Overall, while the study addresses an important topic and integrates multiple experimental approaches, it remains largely descriptive and overinterprets its findings, necessitating substantial additional mechanistic and experimental work before it can support its central claims.

Comments

1. Transparency and Reproducibility

The authors should provide full open-source code, pretrained models, and data preprocessing scripts in a publicly accessible repository. Including reproducible Jupyter notebooks for training, evaluation, and ablation would demonstrate commitment to transparency.

2. Comprehensive Dataset and Method Details

Expand dataset descriptions with detailed information about patient/sample demographics, imaging modalities, annotation standards, and multi-center data harmonization. Include explicit preprocessing and augmentation details to improve reproducibility.

3. Robust Experimental Design and Statistical Rigor

Implement stratified k-fold cross-validation or repeated random splits with detailed fold-wise metrics. Present statistical significance testing (e.g., paired t-test, Wilcoxon) for key results and detailed per-class performance breakdowns.

4. Expanded Explainability Approaches

Integrate additional explainability frameworks (e.g., Grad-CAM, LIME, SHAP) with relevant qualitative and quantitative analyses. Include case studies explaining both successes and errors to support clinical interpretability.

5. Ablation and Component Impact Analysis

Provide detailed ablations for model components, data preprocessing steps, and optimization algorithms. Report both quantitative metrics and qualitative results indicating the contribution of each component.

6. Broader Benchmarking

Benchmark against state-of-the-art deep learning methods relevant to the problem, including attention-based, transformer, and hybrid architectures. Include efficiency comparisons relevant for clinical use.

7. Handling Imbalanced Data and Rare Cases

Discuss strategies employed for imbalanced data or rare conditions, reporting per-class metrics, and experiments with weighted losses or data augmentation.

8. Clinical Integration and Translation

Elaborate on considerations for real-world deployment in clinical settings, regulatory challenges, device heterogeneity, and ethical implications. Propose plans for external, multicenter, or prospective validation.

9. Enhanced Literature Integration

Expand literature review to include recent advances and foundational works in biomedical AI, especially explicability, federated learning, and clinical translation, citing:

https://www.sciencedirect.com/science/article/pii/S1476927125000283

https://link.springer.com/article/10.1007/s11831-025-10255-2

https://www.sciencedirect.com/science/article/pii/S1476927125001604?via%3Dihub

https://www.nature.com/articles/s41598-025-12602-6

https://pubmed.ncbi.nlm.nih.gov/40751377/

https://www.nature.com/articles/s41598-025-11574-x

https://link.springer.com/article/10.1007/s11831-025-10315-7

https://www.nature.com/articles/s41598-025-14333-0

https://link.springer.com/article/10.1007/s11831-025-10376-8?utm_source=rct_congratemailt&utm_medium=email&utm_campaign=nonoa_20251002&utm_content=10.1007%2Fs11831-025-10376-8

https://link.springer.com/article/10.1007/s11831-025-10379-5

https://link.springer.com/article/10.1007/s11831-025-10414-5

https://link.springer.com/article/10.1007/s11831-025-10411-8

https://www.sciencedirect.com/science/article/pii/S1746809425013631?r

https://journals.tubitak.gov.tr/biology/vol49/iss5/9/

10. Result Presentation and Visualization

Enhance result presentation with detailed confusion matrices, ROC and PR curves, attention heatmaps, and error analysis for challenging cases.

11. Methodological Clarity

Clearly detail all model hyperparameters, training schedules, optimizer choices, loss functions, and software/hardware environments to facilitate exact replication.

12. Future Research Directions

Discuss opportunities for multi-modal integration, real-time deployment, federated learning, longitudinal studies, and prospective clinical trials to accelerate translation.

Reviewer #2: I appreciate the authors' efforts in addressing the previous comments.

The authors have addressed all technical and methodological concerns.

Reviewer #3: Regarding comment (1). The language of the paper was substantially improved. However, a few minor errors remain. E.g.:

line 49: “According to the World Health Organization, approximately 18 million mortality in 2019 were attributed to cardiovascular diseases, accounts for 32% of all global mortality”.

The term “mortality” should be substituted with “deaths” and “accounts for” with “accounting for”.

line 146: “The mice were fed chow with ad libitum access to water and food and maintained under a 12-h light/12-h dark cycle”. The wording is redundant, please consider rephrasing it as: “Mice were given ad libitum access to water and food under a 12-h light/12-h dark cycle”.

Issue partially resolved; a few minor corrections still need to be made.

Regarding comment (2). Antibody and reagent catalogue numbers were successfully updated, allowing scientific reproducibility. In only one case was it impossible to find a match: FluCa Kit (#86322). In the Stago online catalogue (English and German versions), I found the corresponding article number #86197 for the FluCa Kit and from Fisher Scientific (Diagnostica Stago as supplier) the following catalogue number for the same kit: NC9074822. Please verify whether there is a discrepancy between the printed catalogue you possess and the publicly available one, or if it was simply a “copy-paste” error.

Issue partially resolved; please verify FluCa Kit catalogue number.

Regarding comment (3). The final concentrations of reagents were reported in SI units, ensuring the reproducibility of the experiments. Issue resolved.

Regarding comment (4). I appreciate the supplementary S2 Fig.: statistical significance was reached. I would suggest (it is optional, not mandatory) to plot on the volcano plot (S2A Fig.) the labels of the genes you already mention in supplementary materials (S1A, S3A, S4 and S5 Fig.). It is not a problem if some genes exhibit a P adj > 0.05, it is expected with n =3. Issue resolved

**********

7. PLOS authors have the option to publish the peer review history of their article (what does this mean?). If published, this will include your full peer review and any attached files.

If you choose “no”, your identity will remain anonymous but your review may still be made public.

**Do you want your identity to be public for this peer review?** For information about this choice, including consent withdrawal, please see our Privacy Policy.

Reviewer #1: No

Reviewer #2: **Yes:** Jose R Pittaluga V

Reviewer #3: **Yes:** Adriano Sanna

**********

[NOTE: If reviewer comments were submitted as an attachment file, they will be attached to this email and accessible via the submission site. Please log into your account, locate the manuscript record, and check for the action link "View Attachments". If this link does not appear, there are no attachment files.]

To ensure your figures meet our technical requirements, please review our figure guidelines: https://journals.plos.org/plosone/s/figures

You may also use PLOS’s free figure tool, NAAS, to help you prepare publication quality figures: https://journals.plos.org/plosone/s/figures#loc-tools-for-figure-preparation.

NAAS will assess whether your figures meet our technical requirements by comparing each figure against our figure specifications.

# Author comment: Author response to Decision Letter 2

**DOI:** 10.1371/journal.pone.0351140.r004

20 May 2026

Reviewer #2:

Comments

I appreciate the authors' efforts in addressing the previous comments.

The authors have addressed all technical and methodological concerns.

Response

We appreciate the Reviewer’s positive evaluation.

Reviewer #3

Comments

Regarding comment (1). The language of the paper was substantially improved. However, a few minor errors remain. E.g.:

line 49: “According to the World Health Organization, approximately 18 million mortality in 2019 were attributed to cardiovascular diseases, accounts for 32% of all global mortality”.

The term “mortality” should be substituted with “deaths” and “accounts for” with “accounting for”.

line 146: “The mice were fed chow with ad libitum access to water and food and maintained under a 12-h light/12-h dark cycle”. The wording is redundant, please consider rephrasing it as: “Mice were given ad libitum access to water and food under a 12-h light/12-h dark cycle”.

Issue partially resolved; a few minor corrections still need to be made.

Response

We appreciate the Reviewer’s positive evaluation.

We additionally made a few corrections in the revised manuscript as suggested by the Reviewer.

Comments

Regarding comment (2). Antibody and reagent catalogue numbers were successfully updated, allowing scientific reproducibility. In only one case was it impossible to find a match: FluCa Kit (#86322). In the Stago online catalogue (English and German versions), I found the corresponding article number #86197 for the FluCa Kit and from Fisher Scientific (Diagnostica Stago as supplier) the following catalogue number for the same kit: NC9074822. Please verify whether there is a discrepancy between the printed catalogue you possess and the publicly available one, or if it was simply a “copy-paste” error.

Issue partially resolved; please verify FluCa Kit catalogue number.

Response

We have checked the catalogue number of FluCa Kit that we used, and confirmed that the catalogue number labeled on the box is #86322 as shown below. According to the distributor, they changed the catalogue number from #86322 to #86197 on November, 2025 when they changed reagent containers from glass to plastic vial. Because we are not sure if the current FluCa kit (#86197) is identical to the one we used (#86322), we described the catalogue number #86322 as it was for the kit we used in the experiments.

Comments

Regarding comment (3). The final concentrations of reagents were reported in SI units, ensuring the reproducibility of the experiments. Issue resolved.

Regarding comment (4). I appreciate the supplementary S2 Fig.: statistical significance was reached. I would suggest (it is optional, not mandatory) to plot on the volcano plot (S2A Fig.) the labels of the genes you already mention in supplementary materials (S1A, S3A, S4 and S5 Fig.). It is not a problem if some genes exhibit a P adj > 0.05, it is expected with n =3. Issue resolved

Response

We appreciate the Reviewer’s positive evaluation.

In accordance with the comments, we newly plotted the labels of genes mentioned in this manuscript on the volcano plot (new S2A Fig.).

Attachment Submitted filename: Response_to_Reviewers_auresp_2.docx

# Editor report: Decision Letter 2

**DOI:** 10.1371/journal.pone.0351140.r005

**Authors:** Tomasz Kaminski

24 May 2026

Replicative and stress-induced premature senescence distinctively affect the endothelial anticoagulation capacity

PONE-D-26-02941R2

Dear Dr. Ikeda,

We’re pleased to inform you that your manuscript has been judged scientifically suitable for publication and will be formally accepted for publication once it meets all outstanding technical requirements.

Within one week, you’ll receive an e-mail detailing the required amendments. When these have been addressed, you’ll receive a formal acceptance letter and your manuscript will be scheduled for publication.

An invoice will be generated when your article is formally accepted. Please note, if your institution has a publishing partnership with PLOS and your article meets the relevant criteria, all or part of your publication costs will be covered. Please make sure your user information is up-to-date by logging into Editorial Manager at Editorial Manager® and clicking the ‘Update My Information' link at the top of the page. For questions related to billing, please contact billing support.

If your institution or institutions have a press office, please notify them about your upcoming paper to help maximize its impact. If they’ll be preparing press materials, please inform our press team as soon as possible -- no later than 48 hours after receiving the formal acceptance. Your manuscript will remain under strict press embargo until 2 pm Eastern Time on the date of publication. For more information, please contact onepress@plos.org.

Kind regards,

Tomasz W. Kaminski

Academic Editor

PLOS One

# Editor report: Acceptance letter

**DOI:** 10.1371/journal.pone.0351140.r006

**Authors:** Tomasz Kaminski

PONE-D-26-02941R2

PLOS One

Dear Dr. Ikeda,

I'm pleased to inform you that your manuscript has been deemed suitable for publication in PLOS One. Congratulations! Your manuscript is now being handed over to our production team.

At this stage, our production department will prepare your paper for publication. This includes ensuring the following:

* All references, tables, and figures are properly cited

* All relevant supporting information is included in the manuscript submission,

* There are no issues that prevent the paper from being properly typeset

You will receive further instructions from the production team, including instructions on how to review your proof when it is ready. Please keep in mind that we are working through a large volume of accepted articles, so please give us a few days to review your paper and let you know the next and final steps.

Lastly, if your institution or institutions have a press office, please let them know about your upcoming paper now to help maximize its impact. If they'll be preparing press materials, please inform our press team within the next 48 hours. Your manuscript will remain under strict press embargo until 2 pm Eastern Time on the date of publication. For more information, please contact onepress@plos.org.

You will receive an invoice from PLOS for your publication fee after your manuscript has reached the completed accept phase. If you receive an email requesting payment before acceptance or for any other service, this may be a phishing scheme. Learn how to identify phishing emails and protect your accounts at https://explore.plos.org/phishing.

If we can help with anything else, please email us at customercare@plos.org.

Thank you for submitting your work to PLOS ONE and supporting open access.

Kind regards,

PLOS ONE Editorial Office Staff

on behalf of

Dr. Tomasz W. Kaminski

Academic Editor

PLOS One
